# Hazard characterization of the mycotoxins enniatins and beauvericin to identify data gaps and improve risk assessment for human health

**DOI:** 10.1007/s00204-025-03988-3

**Published:** 2025-03-26

**Authors:** Anne-Cathrin Behr, Christiane Kruse Fæste, Amaya Azqueta, Ana M. Tavares, Anastasia Spyropoulou, Anita Solhaug, Ann-Karin Olsen, Ariane Vettorazzi, Birgit Mertens, Bojana Zegura, Camille Streel, Dieynaba Ndiaye, Eliana Spilioti, Estelle Dubreil, Franca Maria Buratti, Francesco Crudo, Gunnar Sundstøl Eriksen, Igor Snapkow, João Paulo Teixeira, Josef D. Rasinger, Julie Sanders, Kyriaki Machera, Lada Ivanova, Laurent Gaté, Ludovic Le Hegarat, Matjaz Novak, Nicola M. Smith, Sabrina Tait, Sónia Fraga, Sonja Hager, Doris Marko, Albert Braeuning, Henriqueta Louro, Maria João Silva, Hubert Dirven, Jessica Dietrich

**Affiliations:** 1 Department Food Safety, BfR German Federal Institute for Risk Assessment, Max-Dohrn-Straße 8-10, 10589 Berlin, Germany; 2 NVI Norwegian Veterinary Institute, PO box 64, 1431 Ås, Norway; 3 Department of Pharmaceutical Sciences, UNAV University of Navarra, Pamplona, Spain; 4 INSA National Institute of Health Dr. Ricardo Jorge, Department of Human Genetics and ToxOmics, Centre for Toxicogenomics and Human Health, Nova Medical School/Faculdade de Ciências Médicas, Universida de Nova de Lisboa, Lisbon, Portugal; 5 Laboratory of Toxicological Control of Pesticides, Scientific Directorate of Pesticides’ Control and Phytopharmacy, BPI Benaki Phytopathological Institute, 8 Stefanou Delta Street, Kifissia, Attica Greece; 6 NVI Norwegian Veterinary Institute, PO box 64, 1431 Ås, Norway; 7 Department for Environmental Chemistry and Health Effects, NILU - Climate and Environment Institute, PO Box 100, 2027 Kjeller, Norway; 8 Department of Chemical and Physical Health Risks, Sciensano, Brussels, Belgium; 9 NIB National Institute of Biology, Večna Pot 121, Ljubljana, Slovenia; 10 INRS Institut National de Recherche et de Sécurité Pour La Prévention Des Accidents du Travail Et Des Maladies Professionnelles, Rue du Morvan, CS 60027, 54519 Vandœuvre-Lès-Nancy Cedex, France; 11 Fougères Laboratory, Toxicology of Contaminants Unit, ANSES French Agency for Food, Environmental and Occupational Health and Safety, 35306 Fougères Cedex, France; 12 Mechanisms, Biomarkers and Models Unit, Department Environmental and Health, ISS Istituto Superiore Di Sanità, Viale Regina Elena 299, 00161 Rome, Italy; 13 Faculty of Chemistry, Department of Food Chemistry and Toxicology, UNIVIE University of Vienna, Vienna, Austria; 14 Department of Chemical Toxicology, NIPH Norwegian Institute of Public Health, 0456 Oslo, Norway; 15 Department of Environmental Health, INSA National Institute of Health Dr. Ricardo Jorge, Porto, Portugal; 16 EPIUnit-Institute of Public Health, University of Porto and Laboratory for Integrative and Translational Research in Population Health (ITR), Porto, Portugal; 17 IMR Norwegian Institute of Marine Research, Nordnes, PO box 1870, 5817 Bergen, Norway; 18 Faculty of Pharmacy and Nutrition, Department of Pharmaceutical Sciences, MITOX Research Group, UNAV - University of Navarra, Pamplona, Spain

**Keywords:** Enniatins, Beauvericin, Genotoxicity, Endocrine effects, Immunotoxicology, Toxicokinetics

## Abstract

Enniatins (ENNs) and beauvericin (BEA) are cyclic hexadepsipeptide fungal metabolites which have demonstrated antibiotic, antimycotic, and insecticidal activities. The substantial toxic potentials of these mycotoxins are associated with their ionophoric molecular properties and relatively high lipophilicities. ENNs occur extensively in grain and grain-derived products and are considered a food safety issue by the European Food Safety Authority (EFSA). The tolerable daily intake and maximum levels for ENNs in humans and animals remain unestablished due to key toxicological and toxicokinetic data gaps, preventing full risk assessment. Aiming to find critical data gaps impeding hazard characterization and risk evaluation, this review presents a comprehensive summary of the existing information from in vitro and in vivo studies on toxicokinetic characteristics and cytotoxic, genotoxic, immunotoxic, endocrine, reproductive and developmental effects of the most prevalent ENN analogues (ENN A, A1, B, B1) and BEA. The missing information identified showed that additional studies on ENNs and BEA have to be performed before sufficient data for an in-depth hazard characterisation of these mycotoxins become available.

## Introduction

**Fig. 1:**
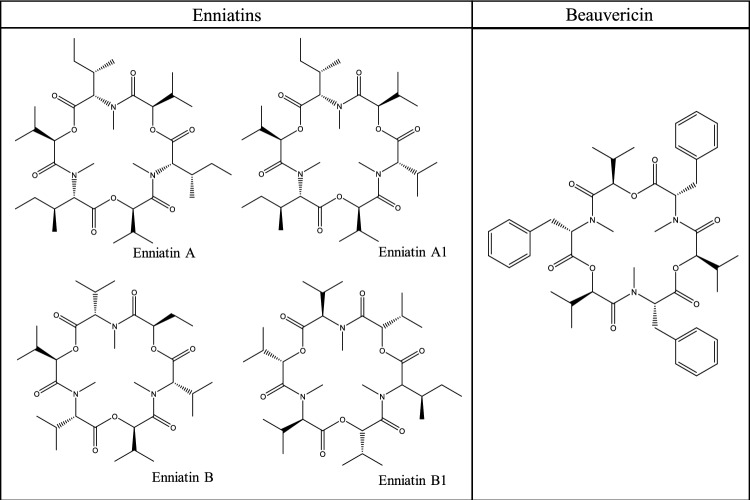
Structure of enniatins and beauvericin

Mycotoxins are secondary metabolites produced by different toxin-producing fungi species, e.g. *Aspergillus* spp., *Fusarium* spp., *Alternaria* spp. and *Penicillium* spp. depending on the prevailing environmental conditions. They may be produced either on field or under transport/storage conditions and are thus mainly found in plant-based foodstuffs like cereals, nuts, oil seeds, and spices. Moreover, the carry-over to food products derived from animals fed with mycotoxin-containing feedstuffs should be considered. Dietary exposure to mycotoxins may result in a wide variety of chronic and sometimes acute health effects for humans and animals (Creppy 2002). Thus, their occurrence in food shall be kept as low as reasonably achievable, and the European Commission has set maximum levels for certain mycotoxins (e.g., aflatoxins, deoxynivalenol, patulin, fumonisins, zearalenone, and ochratoxin A) in certain food- and feedstuffs (2006/576/EC; Regulation 2023/915). In contrast, some mycotoxin groups/compounds are neither regulated nor covered by the analytical methods of routine official controls. These compounds are commonly referred to as “emerging mycotoxins” due to the lack of sufficient data regarding the potential risks they could pose for human health (Vaclavikova et al. 2013). Among the most prominent representatives of “emerging mycotoxins” are ENNs and BEA, which are secondary metabolites of several *Fusarium* spp. (EFSA 2014; Urbaniak et al. 2020). The structurally related compounds are cyclic hexadepsipeptides, consisting of three a-D-hydroxyisovaleric acid (Hiv) residues alternatively linked to three L-configured N-methyl amino acid residues with different side chains specifying the molecule. The structures of BEA and the four ENN representatives (ENN A, A1, B, B1) occurring most frequently in food are shown in Fig. 1 (EFSA 2014).

The amphiphilic ring structure of ENNs and BEA molecules enables them to form transmembrane cation-selective channels, providing them with ionophoric properties, which are considered the main mode of action and responsible for the observed toxicological effects (EFSA 2014). The disturbance of the physiological cellular homeostasis of monovalent and divalent cations such as Na^+^, K^+^ and Ca^2+^ (Kamyar et al. 2004; Kouri et al. 2003) and channelling of alkali ions into liposomes (Benz 1978; Hilgenfeld and Saenger 2005; Lifson et al. 1984) can initiate event cascades leading to cell death. It has been shown that ENNs and BEA induce apoptosis in different cell lines and act as inhibitors of various enzymes, e.g. acyl-CoA:cholesterol acyltransferase, topoisomerases and multidrug resistance proteins (Dornetshuber et al. 2009a, 2009b; Hiraga et al. 2005; Ivanova et al. 2010; Tomoda et al. 1992). Additionally, the substances have antibiotic and antimicrobial properties as common for many mycotoxins, which serve to the fungi as part of their defence system against predators and food competitors. A mixture of ENNs called fusafungine (containing about 2% ENN A, 16% ENN A1, 40% ENN B and 42% ENN B1) was used as pharmaceutical (1% solution for nasal inhalation) in the treatment of upper respiratory infections from 1978 to 2016 in several European countries before it was discontinued by the European Medicines Agency (EMA) due to concerns about the development of allergies (EFSA 2014; EMA 2016; German-Fattal 2001; Lohmann 1988). Moreover, EMA stated that evidence for beneficial effects of fusafungine is weak. ENNs and BEA are occurring worldwide in grain in temperate climate. Under optimal conditions for the producing fungi, high concentrations can be reached in cereals and cereal-based products that form the main dietary source for humans in the Western world. Furthermore, ENNs and BEA may be present in other plant-based products like herbal products, nuts and fruits and legumes, particularly in dried products (EFSA 2014; Gautier et al. 2020; Gruber-Dorninger et al. 2017). ENN B has been detected in human serum samples, proving considerable dietary exposure to humans: In Sweden, ENN B was found in more than 99% of 1100 analysed serum samples (Warensjo Lemming et al. 2020), while all samples (*n = *50) were positive for ENN B in a study from Germany (Osteresch et al. 2017). In 2014, EFSA’s Panel on contaminants in the food chain (CONTAM) published a “Scientific Opinion on the risks to human and animal health related to the presence of BEA and ENNs in food and feed”. The panel concluded that there is no health concern upon acute exposure, but risk from chronic exposure to ENNs and BEA could not be excluded. However, the available in vivo toxicity and toxicokinetic data were insufficient to perform a risk assessment for chronic exposure (EFSA 2014). Thus, further research in terms of hazard characterisation is required for these toxins.

With this review paper, the authors aim to provide an overview of the current published knowledge regarding the toxicokinetics as well as the cytotoxic, genotoxic, immunotoxic, and endocrine effects of ENNs and BEA, both, in vitro and in vivo. In addition, crucial data gaps are identified that currently hinder comprehensive health risk assessments of ENNs and BEA. Apart from BEA, the primary focus of this review is on ENN B, B1, A and A1, since they are the most prevalent and relevant ENNs in food according to EFSA (2014). This work was carried out in the framework of PARC, which includes research activities to close toxicological data gaps for the risk assessment of natural toxins such as the “emerging mycotoxins” (Marx-Stoelting et al. 2023).

## Toxicokinetics

Since EFSA identified missing toxicokinetic data as critical data gap in their Scientific Opinion (EFSA 2014), several studies providing new information have been performed. Most data are available for BEA, ENN B and ENN B1, whereas ENN A and ENN A1 are less investigated.

### Enniatin B (ENN B)

#### Absorption

A study in rats showed about 8% bioavailability of ENN B after a single oral administration of 10 mg/kg body weight (b.w.) fusafungine containing tritiated ENN B in isotonic saline. The radioactivity levels were measured in faeces and urine for 72 h. However, the value might be underestimated since the label had been incorporated into the methyl group of the N-methyl-L-valine (N-Me-Val) moiety, which in in vitro metabolism experiments has been shown to be subject to oxygenation and dealkylation reactions (Faeste et al. 2011; Ivanova et al. 2011). After application of the same dose intratracheally (i.t.), the maximum concentration (C_max_) of 60% of the applied total radioactivity was reached after 0.5 h (Lohmann 1988). The ENN B levels in serum and urine of female Wistar rats receiving one oral dose of an ENN mixture containing 1.03 mg/kg b.w. ENN B in water were below the limit of quantification (LOQ) (2 ng/mL) up to 8 h after application. However, ENN B was detectable in faeces at all sampling time points with a C_max_ of 166 µg/kg after 6 h. Metabolites of ENN B were not analysed in this study (Escriva et al. 2015). A toxicokinetic study in broiler chicken with oral (p.o.) and intravenous (i.v.) application of 0.2 mg/kg b.w. ENN B dissolved in ethanol resulted in an absolute oral bioavailability of 11%. C_max_ in plasma was 1.0 µg/L at 0.3 h (Fraeyman et al. 2016). A pilot study in one pig with oral application of an ENN mixture containing 0.05 mg/kg b.w. ENN B dissolved in 1:50 acetonitrile/water resulted in a C_max_ of 73.4 µg/L after 0.3 h (Devreese et al. 2013). A toxicokinetic study on ENN B in male CD1 mice, applying 30 mg/kg b.w. p.o. in corn oil/6% dimethyl sulfoxide (DMSO) and 1 mg/kg i.v. in saline/6% DMSO resulted in a C_max,plasma_ of 1 µg/mL after 1h, and a plasma half-life (t1/2) of 0.7h (after i.v.). The absolute oral bioavailability was determined as 140%, supposedly because of the used p.o. formulation. Plasma clearance was not reported (Ojiro et al. 2023).

In vitro experiments in Caco-2 cells revealed medium to high transepithelial transport of 1.5 µM ENN B in DMEM/HEPES buffer with 19.5% and 67% absorption after 1 h and 4 h, respectively (Meca et al. 2012). Another study investigated the transmucosal kinetics of fusafungine in porcine buccal mucosa in an ex vivo*-*in vitro diffusion cell system. The ENN mixture, prepared in ethanol/isopropyl myristate, contained in total 1 mg/mL of ENN B, ENN B1, ENN A and ENN A1 (43.8%, 34.4%, 1.8% and 14%, respectively) and in addition small amounts of ENN C, D and E. Measuring the transport efficiencies for up to 8 h, ENN B reached the highest permeability coefficient, local mucosa concentration and estimated plasma concentration in comparison to the other investigated ENNs. In addition, 0.053% of the applied ENN B amount was found in the receptor chamber (Taevernier et al. 2015). The comparably high values for ENN B were probably related to the toxin’s octanol:water partition coefficient of 4.68, which is the lowest amongst the ENNs. This is in line with another study, where ENN B showed the highest permeation ratio in a human skin model compared to ENN B1, A, A1 or BEA (Taevernier et al. 2016b). Cytotoxicity observed in a long-term exposure of carcinoma-derived cell lines to an ENN mixture containing 19% ENN B was significantly reduced when breast cancer resistance protein (ABCG2) or P-glycoprotein (P-gp) were present, indicating that ENNs are substrates for these transport proteins (Dornetshuber et al. 2009b). In vitro degradation experiments showed that ENN B was depleted by 50% in different probiotic bacteria and by 80% in *Saccharomyces cerevisiae* strains after incubation for 48 h at 37 °C (Roig et al. 2013).

#### Distribution

A study with an intraperitoneal (i.p.) injection of 5 mg/kg b.w. ENN B in 10% DMSO in CB-17 scid/scid immunodeficient mice on two consecutive days showed that the toxin was distributed to multiple organs including liver, kidneys, colon, body fat, brain, and muscles after 24 h. The highest ENN B concentrations were detected in liver (2.9 µg/kg), fat (2.5 µg/kg) and colon (0.9 µg/kg). Moreover, ENN B was determinable in serum (0.45 µg/kg), but not in urine (Rodriguez-Carrasco et al. 2016). In Wistar rats administered with 10 mg/kg b.w. fusafungine containing tritiated ENN B in isotonic saline, 0.5% of the total radioactivity was detected in the liver after 124 h, while 0.06% were detected both, in the kidneys and the brain (Lohmann 1988). Several studies were performed in broiler chicken and laying hens with the aim to investigate potential carry-over into muscle meat and egg. In a survey on Finnish poultry meat (*n = *276) and liver (*n = *43) samples, ENN B was found in 0.6% of the samples, and the highest concentration was 2 µg/kg (Jestoi et al. 2007). Chickens fed with a diet containing 12.7 mg ENN B/kg feed had a carry-over of 0.04% and 0.01% into thigh and breast muscles, respectively, after one week. In addition, 20.5 µg/kg ENN B were detected in average in liver and 50 µg/kg ENN B in skin (Emmanuel et al. 2020). In another experiment in the study by Emmanuel et al. (2020), a carry-over rate of 0.1% was determined in eggs of laying hens receiving a multi-mycotoxin diet containing 11.2 mg ENN B/kg feed for two weeks. The eggs contained 15 µg/kg ENN B, which was reduced to 1 µg/kg after 7 days on a mycotoxin-free diet (Emmanuel et al. 2020). The analysis of Finnish egg samples showed higher concentrations of up to 3.8 µg/kg ENN B in the egg yolks compared to whole eggs. This suggests a bioaccumulation of ENN B in the egg yolk, potentially through transportation by lipoproteins (Jestoi et al. 2009). The ENN B plasma levels in chickens receiving a diet containing 2.352 mg/kg ENN B for 21 days were between < 25 and 264 pg/mL, while the ENN B liver levels were < 0.05 to 0.85 ng/g. The carry-over rates from feed into liver tissue were 0.005 to 0.014%. Histopathologic analysis showed no changes in the liver or the intestine, although the proliferation of enterocytes could be inhibited (Fraeyman et al. 2018a). In broiler chickens receiving 0.2 mg/kg b.w. ENN B i.v. or p.o., a volume of distribution (V_D_) of 33.9 L/kg was determined (Fraeyman et al. 2016). A survey on raw and ultra-high temperature-processed (UHT) milk in Poland determined the presence and highest concentrations of ENN B with, respectively, 41% and 59%, and 0.8 µg/kg and 0.6 µg/kg (Pietruszka et al. 2023). Animal liver samples obtained on a local market in Spain contained ENN B at levels up to 1.5 µg/kg in pig, calf and chicken but not in lamb (Castell et al. 2023). ENN B was not transferred from contaminated feed (20 µg/kg) into the fillets of Atlantic salmon *(Salmo salar*) or Sea bream (*Sparus aurata*) at levels above the limit of detection (LOD) (0.1 µg/kg) over an 11- or 18-month trial period, respectively (Nacher-Mestre et al. 2020). The diet-to-organ transfer rate was also low (< 0.01%) in Atlantic salmon smolt fed with 0.3, 5.2, or 83 mg ENNB/kg diet for 69 days (Berntssen et al. 2023). The ability of ENNs to pass through the blood–brain barrier (BBB) was studied in anaesthetised IRC-CD-1 mice after the intrajugular injection of 0.2 mg/kg b.w. of an ENN mixture (dissolved in 6:94 ethanol/lactated Ringer’s solution with 1% BSA) containing 43.8% ENN B. Blood was drawn from the carotid artery after 15 min post-injection, and the ratio of brain to serum concentrations was used to calculate BBB permeability. The influx rate of ENN B was high with 53 µL/(g*min), the distribution in brain tissue considerable with 29 µL/g, and the efflux negligible (Taevernier et al. 2016a).

ENN B was detected in all breast milk samples of new mothers eating a normal varied diet in Austria by using a highly sensitive liquid chromatography–mass spectrometry/mass spectrometry (LC–MS/MS) method. The authors found ENN B concentrations in the range of the LOQ (1.3 ng/L) and up to 8.8 ng/L, indicating chronic low exposure of breastfed infants and young children (Braun et al. 2020). The analysis of different human tissues from forensic autopsies in Spain revealed high ENN B occurrence in all liver samples (mean: 7 µg/kg). Notable levels were also found in brain, kidney, and lung, whereas the body fat contained less ENN B than other ENNs (Castell et al. 2024).

The transport kinetics of 1 µM ENN B across the BBB were examined in vitro using porcine brain capillary endothelial cells in Transwell filter inserts. After 6.5 h, 53% of the apically applied ENN B was transferred to the basolateral compartment. The authors considered that the permeability of ENN B was in the same order of magnitude as for the high-apparent permeability compounds caffeine and diazepam, which are known to reach the brain (Krug et al. 2018).

#### Metabolism

ENN B metabolites were first determined in in vitro experiments before they were confirmed in in vivo studies.

In CB-17 mice treated with 5 mg/kg b.w. ENN B i.p. for 2 days, three phase I metabolites were identified in liver and colon 24 h after the last injection. No metabolites were found in other tissues or serum. Dioxygenated ENN B reached the highest level, followed by mono- and di-N-demethylated ENN B (Rodriguez-Carrasco et al. 2016). In broiler chickens exposed for 8 days to a mixture of mycotoxins in feed containing 12.7 mg/kg ENN B, three hydroxylated and three carboxylated metabolites were identified in liver, and two hydroxylated and one carboxylated metabolites in serum. N-demethylated metabolites were not observed. After a 1-day depletion period with uncontaminated feed, the metabolite levels in liver were still slightly increased, while they decreased in serum (Ivanova et al. 2014). In the same study, eggs of laying hens fed with a similar diet containing 11.2 mg/kg ENN B were analysed. They contained two hydroxylated metabolites after seven and 14 days, as well as after 1 day on control feed. After 3 days of mycotoxin depletion, only the most prevalent hydroxylated ENN B metabolite was still detectable (Ivanova et al. 2014). Four hydroxylated metabolites were identified in the plasma of broiler chickens up to 8h after p.o. or i.v. application of 0.2 mg/kg b.w. ENN B in broiler chickens. Conjugated phase II metabolites were not detectable (Fraeyman et al. 2016). RNA-Seq analysis in the liver of CD1 mice 2h after receiving a single dose 30 mg/kg b.w. showed significantly increased transcript levels of *Cyp7a1*, *Cyp2a12*, *Cyp2b10*, and *Cyp26a1* (Ojiro et al. 2023).

In the first human biomonitoring study, ENN B and in total 12 monooxygenated, N-demethylated or dioxygenated metabolites were determined in the urine of volunteers from southern Italy (Rodriguez-Carrasco et al. 2018).

ENN B metabolites have been identified in in vitro studies by using their exact masses, fragmentation patterns and retention times in high-performance liquid chromatography high-resolution mass spectrometry (HPLC-HRMS/MS) experiments. Moreover, chemical derivatisation analysis has been used to elucidate changed moieties in the ENN B molecule. The first in vitro biotransformation study was performed in rat (RLM), dog (DLM), and human liver microsomes (HLM) with ENN B concentrations ranging from 0.66 to 1.74 µM (Ivanova et al. 2011). ENN B was rapidly metabolised in HLM to 12 metabolites including monooxygenated, N-demethylated and dioxygenated/carboxylated metabolites. The metabolite profiles between species as well as the formation rates for the individual metabolites differed (Faeste et al. 2011). In a study analysing ENN B metabolisation in chicken liver microsomes, only six of the known metabolites including monooxygenated and carboxylated metabolites were observed. Moreover, a novel ENN B metabolite with hydroxylation in one of the methyl groups of the isopropyl sidechain of the N-Met-Val moiety was identified (Ivanova et al. 2014). The catabolic fate of ENN B was investigated in simulated human digestion and colonic fermentation experiments. The study predicted significant degradation of ENN B during the gastrointestinal passage and microbial metabolisation in the colon, resulting into five metabolites arising from oxidation, opening of the depsipeptide ring and subsequent fragmentation of the open molecule (Pallares et al. 2020).

#### Excretion

While ENN B excretion has been studied by monitoring levels in the urine of mice, rats, pigs, and humans, there exists only one in vivo study so far, which has described the elimination kinetics of ENN B, using broiler chickens. In this study, the systemic clearance (CL) in broiler chickens receiving an i.v. dose of 0.2 mg/kg b.w. ENN B was determined as 7.1 L/(h × kg) and t_1/2_ as 3.3 h (Fraeyman et al. 2016). ENN B was not detected in the urine of i.p. dosed CB-17 mice (Rodriguez-Carrasco et al. 2016) or p.o. dosed Wistar rats (Escriva et al. 2015). However, 5.3% of the total ENN B dose was recovered within 24 h from CD1 mice after application of 30 mg/kg b.w. p.o., whereas only 0.02% was found after 1 mg/kg b.w. i.v. (Ojiro et al. 2023).

ENN B was found at high prevalence in the urine of South-Italian volunteers (87% out of 300 samples) with an average concentration of 0.016 µg/L (range: 0.006 to 0.391 µg/L) (Rodriguez-Carrasco et al. 2018). Moreover, ENN B was measured in 14% of the samples (*n = *50) of German volunteers with a mean concentration of 0.012 µg/L (Gerding et al. 2015), and with levels of up to 0.54 µg/L in the urine (*n = *10) of Spanish volunteers (Escriva et al. 2017). Women (26% of a 540 cohort) participating in the Spanish Childhood and Environment Project (INMA) had creatinine-adjusted ENN B urine levels in the range of 1 to 40 µg/g (Dasi-Navarro et al. 2024). In comparison, the occurrence of urinary ENN B was much lower in study cohorts from Bangladesh and Haiti, which was assumed to result from the different diets in these regions (Escriva et al. 2017). Occupational exposure to mycotoxins during swine production was determined by analysing the urinary levels of workers (*n = *25). ENN B was detected in 4% of the urine samples (Viegas et al. 2019). ENN B was detected in the urine of Spanish volunteers participating in a 24h intervention study comparing conventional and organic diets with a median concentration of 2.4 µg/g creatinine (Gallardo-Ramos et al. 2024). Plasma samples collected from a male Chinese cohort had an incidence rate of 77% for ENN B, indicating chronic exposure (Ning et al. 2024).

In in vitro assays performed under the conditions of linear kinetics, the intrinsic clearances (CL_int_) of ENN B in HLM, RLM and DLM were determined as 1.13, 1.16 and 8.23 L/(h*kg), respectively, and used to predict in vivo blood clearances (CL_b_) as 0.63, 1.57 and 1.67 L/(h*kg), respectively, by applying the well-stirred liver model without consideration of the fraction unbound. The predicted maximum oral bioavailabilities (f_max_) were 63%, 20% and 55%, respectively (Faeste et al. 2011).

### Enniatin B1 (ENN B1)

#### Absorption

ENN B1 levels in the serum and urine of female Wistar rats dosed with a single oral application of an ENN mixture containing 1.41 mg/kg b.w. ENN B1 in water were below the LOQ (2 ng/mL) up to 8 h post application. However, in faeces a maximum concentration of 33.4 ng/g was measured after 6h (Escriva et al. 2015). A toxicokinetic study in broiler chickens with p.o. or i.v. application of 0.2 mg/kg b.w. ENN B1 dissolved in ethanol showed that ENN B1 was poorly absorbed, resulting in an absolute oral bioavailability of 5%. The plasma C_max_ of 1.4 ng/mL was reached after 0.63h (Fraeyman et al. 2016). In a pilot study, one pig was treated with an ENN mixture containing 0.05 mg/kg b.w. ENN B1 dissolved in 1:50 acetonitrile/water. A C_max_ of 35.2 ng/mL was determined after 0.3h (Devreese et al. 2013). In a follow-up study elucidating the toxicokinetic parameters of ENN B1, five pigs were administered with 0.05 mg/kg b.w. in ethanol/water by oral gavage and by i.v. injection. The absolute oral bioavailability was 91%, and the plasma C_max_ was 29.9 ng/mL after 0.24 h (Devreese et al. 2014).

In vitro experiments in Caco-2 cells showed that 23.4% of 1.5 µM ENN B1 were transported from the apical to the basolateral compartment after 1 h, and 67% after 4 h. Basolateral-to-apical transport was not measured (Meca et al. 2012). However, a separate study using 4.8 µM ENN B1 in Caco-2 cells found that the basolateral-to-apical transport was 6.7 times higher than the apical-to-basolateral transport. It was further shown in Caco-2 and MDCK type II cells that ENN B1 is a substrate of P-gp, indicating an active outward transport of the respective intestinal/kidney cells (Ivanova et al. 2010). This was confirmed by a transport study in various human carcinoma-derived cell lines, where an ENN mixture containing 54% ENN B1 was found to be a substrate of multi-drug resistance proteins such as P-gp (Dornetshuber et al. 2009b). The ex vivo examination of the transmucosal transport of 1 mg/mL fusafungine containing 34% ENN B1 in porcine buccal mucosa showed that 0.02% of the applied effective ENN B1 dose was cumulatively found in the receptor chamber (Taevernier et al. 2015). Using the same fusafungine mixture in a human skin model, ENN B1 had the second highest permeation coefficient after ENN B (Taevernier et al. 2016b). In vitro degradation experiments showed that there was a 70% reduction of ENN B1 levels after incubation for 48 h at 37 °C with different probiotic bacteria and a reduction of 85% with *Saccharomyces cerevisiae* strains under the same conditions (Roig et al. 2013).

#### Distribution

Emmanuel et al. (2020) performed two poultry experiments, where the transfer of ENN B1 into tissues of broiler chickens and eggs of laying hens fed with mycotoxin-contaminated feed, including 4.06 mg/kg ENN B1, was examined for 2 weeks. Broiler chickens had detectable ENN B1 levels in meat, with carry-over rates of 0.04% into thigh and 0.03% into breast muscles. The carry-over rate into the liver was 0.1% with 3.8 µg/kg as the highest concentration, which was eliminated within 3 days. The highest concentration in skin was 15 µg/kg ENN B1, which was eliminated within two weeks to levels close to the LOQ (1 µg/kg). The carry-over rate into the skin was 0.4%. The eggs of laying hens fed with feed containing a mycotoxin mixture including 3.6 mg/kg ENN B1 had a carry-over rate of 0.05%. Detectable levels of ENN B1 were found after 3 days, which were depleted after 10 days (Emmanuel et al. 2020). A Finnish survey found ENN B1 in 3% of turkey and broiler meat and liver samples with levels below the LOQ (< 1.12 µg/kg) (Jestoi et al. 2007). In Finnish whole egg samples, ENN B1 occurred regularly at levels close to the LOQ (1.12 µg/kg) (Jestoi et al. 2009). However, in an Algerian study on chicken receiving up to 45 µg/kg ENN B1 in the diet, levels above the quantification level (< 0.8 µg/kg) were not detected in the eggs, which could have resulted from the addition of mycotoxin binders to the feed (Laouni et al. 2024). A study on ENN B1 toxicokinetics in broiler chickens showed that after application of 0.2 mg/kg b.w. ENN B1 dissolved in ethanol i.v. or p.o., the V_D_ was 25.1 L/kg (Fraeyman et al. 2016). In a pilot study with i.v. application of 0.05 mg/kg ENN B1 b.w. in ethanol/water to one pig, the V_D_ was determined as 0.7 L/kg (Devreese et al. 2014).

In Austria, low levels of ENN B1 were detected in breast milk samples of mothers with a normal, varied diet. Concentrations ranged from below the LOQ (1.0 ng/L) to levels slightly above (up to 1.9 ng/L) (Braun et al. 2020). Human tissues obtained from forensic autopsies in Spain showed considerable ENN B1 levels in liver (88% of the samples, mean: 1.3 µg/kg). Additionally, ENN B1 was detected in brain, lung, kidney and fat (mean: 1.8 µg/kg) (Castell et al. 2024).

A study on transport kinetics of ENN B1 across the BBB using porcine brain capillary endothelial cells showed that after 6.5 h, 44% of the applied 1 µM ENN B1 was transferred from the apical to the basolateral compartment. The maximum transport was 55%, noticeably lower than the transport for ENN B (Krug et al. 2018).

#### Metabolism

ENN B1 biotransformation products were determined first in in vivo studies by comparison to already known ENN B metabolites.

In the toxicokinetic study in broiler chickens, two mono-oxygenated, one dioxygenated and one dehydrogenated metabolite were discovered in the plasma already 5 min after i.v. administration. Glucuronidated or sulphated phase II metabolites were not found (Fraeyman et al. 2016). The pig plasma samples from Devreese et al. (2014) were subsequently analysed for metabolites and compared to in vitro results obtained with minipig and slaughter swine liver microsomes. Six phase I metabolites, resulting from hydroxylation, carbonylation, carboxylation, and oxidative demethylation were detected in the in vivo samples, whereas 11 metabolites were produced in vitro. Carbonylated ENN B1 represented the main in vivo metabolite. It appeared that metabolite formation was increased when ENN B1 was absorbed from the gut, which might indicate pre-systemic metabolism after oral uptake (Ivanova et al. 2017).

The presence of ENN B1 and its phase I metabolites were surveyed in 300 urine samples from volunteers in southern Italy. ENN B1 was detected in 94% of the urine samples with levels ranging from 0.007 to 0.429 ng/mL. In total, 11 metabolites were identified, which were products of oxidative demethylation, hydroxylation, carbonylation, and carboxylation reactions. The hydroxylated metabolites (in 78% of the samples) and carbonylated metabolites (in 66% of the samples) were most prevalent. Demethylated and oxidated metabolites were found in 5% of the samples (Rodriguez-Carrasco et al. 2020).

An in vitro study using HLM predicted the production of the same 11 metabolites that were subsequently found in the human urine samples from Italy (Ivanova et al. 2019). The in vitro study included experiments with recombinant CYP3A4 and suggested that this enzyme plays a major role in the human metabolism of ENN B1. Moreover, the metabolisation rate of ENN B1 was considerably decreased in the presence of the mycotoxin deoxynivalenol (DON), indicating an impact of co-occurring substances on ENN B1 metabolism (Ivanova et al. 2019).

#### Excretion

In a rat study, where 1.41 mg/kg b.w. ENN B1 in water was administered p.o., the toxin was found in faeces (maximum level 33.35 µg/kg after 6 h), but not in urine (Escriva et al. 2015). Elimination parameters of ENN B1 were determined in chicken and pig studies after i.v. application. The plasma clearance and half-life in chickens after receiving 0.2 mg/kg b.w. ENN B1 in ethanol was CL = 6.63 L/(h × kg) and t_1/2_ = 2.6h (Fraeyman et al. 2016). In pigs receiving 0.05 mg/kg b.w. ENN B1 in ethanol/water the clearance was 1.91 L/(h×kg) with a half-life of 1.1 h (Devreese et al. 2014).

In epidemiological studies from southern Italy and Spain, ENN B1 was detected in urine samples of the volunteers (Escriva et al. 2017; Rodriguez-Carrasco et al. 2020). In the study from Escriva et al. (2017), the maximum ENN B1 concentration was 0.34 µg/L. In the Spanish INMA-cohort, 7% of the woman had creatinine-adjusted ENN B1 urine levels of 0.5 to 14.4 µg/g (Dasi-Navarro et al. 2024).

The kinetic parameters of ENN B1 were predicted by in vitro-to-in vivo extrapolation based on substrate depletion assays in HLM. The predicted CL_b_ was 0.77 L/(h×kg) and the predicted f_max_ was 45%, which was slightly lower than for ENN B (Ivanova et al. 2019).

### Enniatin A (ENN A)

#### Absorption

Uptake and distribution of ENN A were determined in a 28-day study in Wistar rats (*n = *5) with naturally contaminated feed containing 465 mg ENN A/kg feed. The daily intake of ENN A was estimated as 20.9 mg/kg b.w. per day. At the termination of the feeding study, the serum contained 5.0 µg/mL ENN A. Considerable levels of ENN A were found throughout the gastrointestinal tract, i.e., 9.6 µg/g in the jejunum, 1.3 µg/g in the duodenum, 7.3 µg/g in the colon and 4.6 µg/g in the stomach, indicating incomplete absorption (Manyes et al. 2014). In a follow-up study, ENN A concentrations were measured in the serum of Wistar rats during a 28-day study period. Exposure to 465 mg ENN A/kg feed led to an increase in the mean serum concentrations during the study period: 1.9 µg/mL were reached after two weeks, 2.2 µg/mL after 3 weeks and 4.8 µg/mL after 4 weeks, indicating accumulation of the toxin in the body. ENN A was not detected in urine or faeces during the feeding trial (Juan et al. 2014). The analysis of samples from female Wistar rats dosed with one oral dose of an ENN mixture containing 1.19 mg/kg b.w. ENN A in water showed that the levels in serum and urine were below the LOQ (10 ng/mL) up to 8 h post application. The concentration in faeces was 10.5 ng/g after 6h, which was significantly lower than for ENN B and ENN B1 (Escriva et al. 2015). A toxicokinetic study in rats was performed by administrating 5 mg/kg b.w. ENN A p.o. (in 15% DMSO in corn oil) and 0.4 mg/kg b.w. ENN A i.v. (in DMSO/polyethylene glycol/ethanol/saline 40/20/20/20). The C_max_ of 116 µg/L was reached after 4 h. The absolute bioavailability was determined to be 47% (Bhateria et al. 2022). A toxicokinetic study in one pig with oral application of an ENN mixture containing 0.05 mg/kg b.w. ENN A dissolved in 1:50 acetonitrile/water determined a C_max_ of 6.8 µg/L in plasma after 0.5 h (Devreese et al. 2013). Compared to ENN B and ENN B1, the C_max_ was much lower and reached at a later time point, indicating less efficient and slower absorption of ENN A in pigs.

Transport studies in Caco-2 cells revealed that 20% of 1.5 µM ENN A were transferred from the apical to the basolateral compartment during 1 h, and 77% after 4 h, suggesting a higher transport efficiency as compared to ENN B and ENN B1 (Meca et al. 2012). Studies in different human carcinoma-derived cell lines with an ENN mixture containing 3% ENN A measured ABC transporter-mediated intracellular transport of ENN A using LC–MS/MS analysis, showing that the toxin is a substrate of multi-drug resistance proteins such as P-gp (Dornetshuber et al. 2009b). When the transmucosal transport of 1 mg/mL fusafungine containing 2% ENN A was examined ex vivo in porcine buccal mucosa cells, the concentration in the receptor chamber was below the LOD of 15 ng/L (Taevernier et al. 2015). Using the same fusafungine mixture in a human skin model, ENN A had the lowest permeation coefficient of all ENNs included in the study (Taevernier et al. 2016b).

#### Distribution

In a 28-day study in rats using naturally contaminated feed (465 mg ENN A/kg), the highest ENN A concentration was detected in the liver (22.7 µg/g) at the end of the exposure period, while the ENN A levels in serum and different parts of the intestinal tract were much lower. Other tissues were not analysed (Manyes et al. 2014). In a kinetic study in rats, V_D_ of 9.1 L/kg was determined after i.v. application of 0.4 mg/kg b.w. ENN A. Additional in vitro experiments showed that plasma protein binding was very high, reaching 99% in rat plasma and 99% in human plasma (Bhateria et al. 2022). In a Finnish study on the occurrence of ENNs in turkey, broiler meat and liver samples, ENN A was found in 0.3% of the samples at levels below the LOQ (< 0.03 µg/kg) (Jestoi et al. 2007). ENN A was not detectable in any whole egg sample in a Finnish survey on laying hens (Jestoi et al. 2009). Emmanuel et al. (2020) investigated the carry-over of ENNs to tissues of broiler chickens and eggs of laying hens after two weeks of exposure to feed containing 28 µg/kg ENN A and found no detectable levels in the liver, meat, skin or the eggs (Emmanuel et al. 2020).

ENN A was detected in trace amounts (below the LOQ of 1 ng/L) in 5% of the breast milk samples obtained from new mothers on a normal varied diet in Austria (Braun et al. 2020). Human tissues obtained from forensic autopsies in Spain showed the highest ENN A levels in lipid-rich tissues such as the body fat (mean 4.7 µg/kg) and the lung (mean: 0.6 µg/kg), whereas the concentrations in liver and kidney were low (Castell et al. 2024).

#### Metabolism

In a 28-day rat study with naturally contaminated feed containing 465 mg/kg ENN A, two products of intestinal microbial metabolism were detected in the duodenum and colon. The degradation products included ENN A minus an isoleucine and ENN A minus a hydroxyvaleric acid unit, with concentrations of 89.7 ± 3.2 and 123.55 ± 4.1 mg/L in duodenum digesta, respectively. In the duodenum, two adducts of ENN A with macronutrients originating from feed were found: ENN A + 2 glucose—H_2_0 (196.7 mg/L) and ENN A + 4 glucose (149.4 mg/L). Furthermore, an adduct of ENN A + 2 glucuronic acid (122 mg/L) was detected, which could be a potential product of phase II metabolism. In serum, the adduct ENN A + 2 glucose (66.1 mg/L) was identified (Manyes et al. 2014). The quantitative analysis of ENN A and the description of metabolites in this study is, however, underreported, and the dimension of the detected concentrations (mg/L) appears to be too high by a factor of thousand.

Using recombinant human CYPs, the contributions of the individual enzymes to ENN A biotransformation were determined in vitro as CYP3A4 (74%), CYP1A2 (14%), CYP2C9 (6%) and CYP2E1 (6%) (Bhateria et al. 2022).

Metabolites of ENN A from hepatic metabolism have not been described so far.

#### Excretion

In a toxicokinetic rat study from Bhateria et al. (2022), the clearance was determined as 3.3 L/(h*kg), and the half-life was 1.7 h after i.v. application of 0.4 mg/kg b.w. ENN A (Bhateria et al. 2022). After p.o. application of 1.19 mg/kg b.w. ENN A in water to rats, the unchanged toxin was found in faeces (maximum level 10.5 µg/kg at 6h), but not in urine (Escriva et al. 2015).

ENN A was detected in one urine sample of ten Spanish volunteers at a concentration below the LOQ (0.5 µg/L) (Escriva et al. 2017).

An in vitro study using RLM and HLM predicted in vivo blood clearances of 3.3 L/(h*kg) and 1.1 L/(h*kg), respectively, without consideration of the fraction unbound in blood (Bhateria et al. 2022).

### Enniatin A1 (ENN A1)

#### Absorption

ENN A1 levels determined in serum and urine of rats orally exposed to an ENN mixture in water containing 2.16 mg/kg b.w. ENN A1 were below the LOQ (5 ng/mL) up to 8 h after application. The concentration in faeces was 8.1 µg/kg after 6 h, which was the lowest level of all measured ENNs (Escriva et al. 2015). Oral application of an ENN mixture containing 0.05 mg/kg b.w. ENN A1 dissolved in 1:50 acetonitrile/water to one pig resulted in a C_max_ of 11.6 µg/L in plasma after 0.3h. The C_max_ was significantly lower as compared to ENN B and ENN B1, but higher than for ENN A (Devreese et al. 2013).

Transport studies in Caco-2 cells revealed that 22% of 1.5 µM ENN A1 were transferred from the apical to the basolateral compartment after 1 h, and 70% after 4 h (Meca et al. 2012). By LC–MS/MS analysis, it was shown in different human carcinoma-derived cell lines incubated with an ENN mixture containing 20% ENN A1 that the toxin is a substrate of multi-drug resistance proteins such as P-gp (Dornetshuber et al. 2009b). When the transmucosal transport of 1 mg/mL fusafungine containing 14% ENN A1 was examined ex vivo in porcine buccal mucosa cells, 0.008% of the applied ENN A1 dose were cumulatively found in the receptor chamber (Taevernier et al. 2015). Using the same fusafungine mixture in a human skin model, ENN A1 showed a low permeation coefficient, comparable to that of ENN A (Taevernier et al. 2016b).

#### Distribution

In a Finnish study investigating the occurrence of ENNs in turkey and broiler meat and liver samples, ENN A1 was detected in 0.6% of the samples at concentrations below the LOQ (0.42 µg/kg) (Jestoi et al. 2007). ENN A1 was not found in any whole egg sample in a Finnish survey (Jestoi et al. 2009). In an experiment investigating the carry-over of mycotoxins to the liver, meat and skin after a 2-week exposure to mycotoxin-contaminated feed containing 491 µg/kg ENN A1, no detectable levels were found (Emmanuel et al. 2020). Emmanuel et al. (2020) also investigated the transfer from feed containing amongst other toxins 440 µg/kg ENN A1 into eggs and did also not find toxin at levels above the LOD (0.7 µg/kg).

ENN A1 was detected in trace amounts (below the LOQ of 1.8 ng/L) in only one breast milk sample of new mothers on a normal, varied diet in Austria (Braun et al. 2020). In human tissues obtained from forensic autopsies in Spain ENN A1 levels were highest in the body fat (mean: 1 µg/kg), followed by liver and lung (Castell et al. 2024).

#### Metabolism

Metabolites of ENN A1 from hepatic or microbial metabolism have not been described so far.

#### Excretion

ENN A1 was found in the faeces (maximum level 8.1 µg/kg after 6 h), but not in urine of rats administered p.o. 2.16 mg/kg b.w. ENN A1 in water (Escriva et al. 2015).

ENN A1 was detected at concentrations close to the LOQ (0.3 µg/L) in the urine of Spanish volunteers (Escriva et al. 2017). Data on plasma half-life or clearance are not available for ENN A1.

### Beauvericin (BEA)

#### Absorption

Mei et al. (2009) administered rats with 0.5, 1.0 or 2.0 mg/kg b.w. BEA p.o. in 0.5% methylcellulose and determined plasma C_max_ as 3.4, 5.4 and 13.9 mg/L at 4.1, 4.3, and 5.4 h, respectively. Dose-normalised, the C_max_ were 6.80, 5.40, and 6.95 kg b.w./L, indicating dose linearity (Mei et al. 2009). In a toxicokinetic study in rats with BEA application of 0.5 mg/kg i.v. and 2 mg/kg b.w. p.o., the absolute bioavailability was 29%. The C_max_ of 41.6 µg/L was reached after 1 h (Yuan et al. 2022). The formulation was not presented in the method description. By comparing the C_max_ of both rat studies, it becomes apparent that the given unit for plasma concentrations (mg/L) in the study of Mei et al. (2009) might be wrong. In a toxicokinetic study in one pig with an orally applied ENN mixture and 0.05 mg/kg b.w. BEA dissolved in 1:50 acetonitrile/water, it was not possible to measure a plasma concentration profile for BEA. The plasma concentrations were above the LOQ (0.2 µg/L) only at 0.5 h and 0.67 h after the application, resulting in a plasma C_max_ of 0.82 µg/L at 0.67 h (Devreese et al. 2013).

Unidirectional transport through Caco-2 cells was assessed with 1.5 µM BEA resulting in a transfer rate of 16% after 1h and 54% after 4h (Prosperini et al. 2012). A study in carcinoma-derived cell lines indicated that BEA is a substrate of transmembrane transport proteins such as P-gp, but to a lower extent than the other tested ENNs (Dornetshuber et al. 2009b). In a human skin model, BEA showed a lower permeation coefficient than the tested ENNs and appeared to accumulate in the skin (Taevernier et al. 2016b).

#### Distribution

A study determining the tissue distribution in mice administered i.p. with 5 mg/kg b.w. BEA dissolved in 10% DMSO on three consecutive days showed that the toxin was detectable in liver, kidneys, colon, body fat, brain, and muscles after 24 h. The highest BEA concentrations were detected in liver (41.7 µg/kg), fat (33 µg/kg) and colon (25.4 µg/kg) at levels ten times higher than those for ENN B. BEA was also determinable in serum (1.3 µg/kg), but not in urine (Rodriguez-Carrasco et al. 2016). In a toxicokinetic rat study with application of 0.5 mg/kg b.w. BEA i.v. the V_D_ was determined as 5.9 L/kg (Yuan et al. 2022). BEA was stable in the plasma of different species and the plasma protein binding was high. The unbound fraction was 0.1% in human plasma, 0.07% in Sprague Dawley rat plasma, 0.06% in CD-1 mouse plasma, 0.08% in Beagle dog plasma and 0.09% in Cynomolgus monkey plasma (Yuan et al. 2022). The diet-to-organ transfer rate was low (< 0.01%) in Atlantic salmon smolt fed with 0.3, 4.8, or 46 mg BEA/kg feed for 3 months (Berntssen et al. 2023). A survey on raw and UHT milk in Poland determined the presence of BEA with 20% and 33%, and the highest concentrations as 6.2 µg/kg and 1.9 µg/kg, respectively (Pietruszka et al. 2023). The animal liver samples obtained on a local market in Spain contained BEA at levels up to 1.1 µg/kg in calf and 0.7 µg/kg in lamb, but not in pig and chicken (Castell et al. 2023).

In a study analysing human breast milk from new mothers on a normal varied diet in Austria, BEA was found in all samples at concentrations ranging from below the LOQ (0.3 ng/L) to 2.9 ng/L (Braun et al. 2020). BEA was detected only in the body fat (mean: 0.5 µg/kg) and brain amongst human tissues obtained from forensic autopsies in Spain (Castell et al. 2024).

#### Metabolism

Data on BEA metabolites produced by hepatic biotransformation in vivo are not available.

In vitro experiments using HLM and RLM investigated the involvement of phase I enzymes in BEA metabolism. By performing competitive inhibition studies, it was found that BEA is probably metabolised by CYP3A4/5 and CYP2C19 in humans, and by CYP3A1/2 in rats (Mei et al. 2009). Yuan et al. (2022) used RLM, DLM, HLM, mouse (MLM) and Cynomolgus monkey (CLM) microsomes as well as cryopreserved hepatocytes of the same species to determine the affinity of BEA to specific CYP enzymes. It was shown that BEA was competitively inhibiting the enzymatic reactions catalysed by CYP3A4 and CYP2C19 in HLM and reactions catalysed by CYP3A1/2 in RLM. The effects on human CYP1A2, CYP2D6 and CYP2C9 (half-maximal inhibitory concentration (IC_50_) > 10 µM) were significantly weaker. In further experiments, the formation of BEA metabolites was observed. In total, 15 metabolites were found in CLM and 13 in the other species. The metabolites were identified by liquid chromatography–ultraviolet mass spectrometry (LC–UV–MS) as mono-, di-, and tri-oxygenated and N-demethylated BEA. Moreover, phase II reaction conjugates with glutathione and cysteine were identified (Yuan et al. 2022).

#### Excretion

The plasma half-lives after oral administration of 0.5, 1 or 2 mg/kg b.w. BEA to rats were determined as 2.9, 3.6, and 3 h, respectively (Mei et al. 2009). After intravenous application of 0.5 mg/kg b.w. to rats, the plasma half-life was 5.1 h. The plasma clearance was determined as 1.43 ± 0.26 L/(h × kg). The oral application of 2 mg/kg b.w. BEA resulted in a plasma half-life of 5.9 h (Yuan et al. 2022).

BEA was not detected in the urine of ten Spanish volunteers (LOD = 0.3 µg/L) (Escriva et al. 2017). However, 69% of the plasma samples collected from a male Chinese cohort contained BEA, indicating chronic exposure (Ning et al. 2024).

The aforementioned studies from Mei et al. (2009) and Yuan et al. (2022) also examined the elimination kinetics of BEA in in vitro assays with microsomes and cryopreserved primary hepatocytes of different species. Mei et al. (2009) determined the Michaelis constant (K_m_) and maximum enzyme velocity (V_max_) using HLM for incubations with start concentrations ranging from 40 to 1000 nM as K_m_ = 0.6 ± 0.1 µM and V_max_ = 21 ± 3 nM/(min*mg protein). Thereof, the intrinsic clearance CL_int,mic_ in HLM was calculated as 38 ± 8 mL/(min × mg protein). However, slightly differing values were given in the discussion of that article and it appears that the unit dimension of the volume should be µL (Mei et al. 2009).

In comparison, Yuan et al. (2022) determined CL_int,mic_ of 1 µM BEA in HLM, RLM, DLM, MLM and CLM as 30.9, 54.2, 112.7, 228.7 and 191.1 µL/(min × mg protein), respectively (Yuan et al. 2022). Thus, the CL_int,mic_ in HLM would be similar in both in vitro studies, if the unit for volume was corrected in Mei et al. (2009). Based on the microsomal clearances, Yuan et al. (2022) calculated the intrinsic liver clearances in humans, rats, mice, dogs and monkeys as CL_int,liver_ = 27.8, 97.6, 446.2, 329.4 and 258.0 mL/(min × kg b.w.), respectively. When cryopreserved primary hepatocytes of the same species were used, the intrinsic liver clearances were calculated as CL_int,liver_ = 46.3, 85.7, 229.7, 612.5 and 178.0 mL/(min × kg b.w.), respectively. They are thus approximately in the same range as the values determined in the microsomal experiments. In vitro-to-in vivo prediction of blood clearances was not performed (Yuan et al. 2022).

### Summary

Compared to regulated mycotoxins, the toxicokinetic data for ENNs and BEA are scarce. As a result, it is challenging to perform adequate hazard characterisation for the assessment of human risk. Data are more abundant for the most common ENNs, specifically ENN B and ENN B1, while there is comparatively less research on ENN A, ENN A1, and BEA.

There is a wide range regarding the observed absorption of the lipophilic hexapeptidic molecules from the gastrointestinal tract into the systemic circulation. The available in vivo data suggested an oral bioavailability for ENNs in the range of 5 to 90% in the species investigated. The bioavailability of BEA is apparently lower than for ENNs, considering the results from a pilot study in one pig, which had been applied with an ENN mixture and BEA. The maximum plasma concentration peaks within 0.5 h after oral uptake. In vitro transport studies indicated decreasing absorbability in the order ENN B > ENN B1 > ENN A1 > ENN A > BEA. The limited intestinal absorption could be one reason why there is a lack of correlation between the in vitro and in vivo toxicity of ENNs and BEA.

Distribution of the ENNs and BEA into different body tissues including liver, body fat, kidneys, brain, skin and muscles occurs rather rapidly. The toxins bind to plasma proteins, resulting in a low fraction unbound, which is below 0.6% for ENNs, and below 0.1% for BEA. Carry-over occurs into human breast milk and into eggs. There are, however, differences in the transport efficiencies, equivalent to the order of absorbability, with ENN B demonstrating the highest carry-over rates. In vitro transport experiments with colon and brain cell lines confirm the relatively high transmembrane permeability of ENN B. Intracellular levels are nevertheless limited, because the toxins are apparently substrates of transport proteins such as P-gp. Considering the distribution characteristics of the ENNs and BEA, the V_D_ can be expected to be considerably high, which has been shown for ENN B1 in chicken and ENN A in rats, but not for ENN B1 in pig.

Metabolism of ENN B, ENN B1, ENN A and BEA is extensive in the species investigated and catalysed by phase I enzymes, especially CYP3A. Data for ENN A1 are not available. Major metabolites, identified in several species, are hydroxylated, mono- and dioxygenated, mono- and di-N-demethylated or carboxylated. ENN metabolites are present in the plasma, liver and gut of experimental animals, in eggs of laying hens, and in human urine samples. Species differences regarding the metabolite profiles can be observed. Information on BEA metabolism is restricted to in vitro biotransformation experiments. There is good agreement between ENN metabolites determined in vitro and in vivo, but the metabolite ratios differed suggesting potential pre-systemic metabolism. This is supported by observed microbial metabolism of ENN B and ENN A, which produced novel metabolites with opened ring structure, and three conjugated metabolites.

Excretion kinetics of the ENNs and BEA is not well documented. According to the available in vivo data for ENN B and ENN B1 in broiler chicken, for ENN B1 in pig and ENN A in rat, the systemic clearances are close to the hepatic blood flow. In contrast, the clearance of BEA in rat was much lower. Plasma half-lives are consistently about 3 h for the species and toxins studied in vivo. Considerable amounts of orally applied ENNs and BEA are excreted unabsorbed in faeces. From the systemic circulation, the toxins are eliminated by hepatic metabolism and urinary excretion.

## General toxicity in vitro and in vivo

There are numerous in vitro studies investigating the cytotoxicity of ENNs and BEA. They have been carried out for individual ENNs and BEA as well as for mixtures of ENNs, since these mycotoxins might occur simultaneously in food and feed products. Toxic effects of ENNs and BEA in vivo have been described in literature, revealing a diverse range of outcomes observed both within studies and across different species.

### In vitro studies

#### Individual toxins

Cytotoxicity data are available for ENN A, A1, B, B1, and BEA as well as for the rarer ENNs A2, B2, B3, B4, M, I, and J3 (Table 1). The toxicity of these toxins has been tested in a wide range of human cell lines, particularly those originating from intestine (Caco-2, HCT15, HCT116, and HT-29), lung (A549, BEAS-2B, and MRC-5), and liver (HepG2). Moreover, cell lines from skin, ovary, brain, blood cells, mammary tissue, uterus, kidney, umbilical cord, stomach, and bone marrow have been used. Additionally, those mycotoxins’ toxicity was studied in cell lines derived from various animals, including rodents, pigs, dogs, calf, monkey, fish, and insects. However, the IC_50_ values estimated from MTT assays might be underestimated, as it is shown that substrates/inhibitors of efflux pumps, such as ENNs and BEA, interfere with this viability assay (Dornetshuber et al. 2009b; Ivanova et al. 2010; Vellonen et al. 2004).

**Table 1:** In vitro cytotoxicity studies on beauvericin and enniatins

Cell model	Tissue	Exposure conditions (mycotoxin, concentration, duration)	Assa	Effect, half-maximal inhibitory concentration (IC_50_)	References
Beauvericin					
A375SM	Skin	0.08–20 µM 72h	MTT	3.0 µM	Lim et al. (2020)
A549	Lung	0.03–10 µM 72 h	SRB	1.4 µM	Lee et al. (2008)
A549	Lung	1–10 µM 24h	MTT	~ 65% viable cells at 3 µM *	Lu et al. (2016)
BEAS-2B	Lung	0–100 µM 48h	Resazurin-based toxicity assay	6.3 µM	Olleik et al. (2019)
BFU-E	Blood	6.4 nM–64 µM 12–14 days	Clonogenic assay	3.7 µM	Ficheux et al. (2012)
C6	Brain	0–25 µM 4h, 24h	MTT	4h: 7.5 µM 24h: 1.0 µM	Watjen et al. (2014)
Caco-2	Intestine	0.6–30 µM 24 h, 48 h	MTT	24 h: 20.6 µM 48 h: 12.8 µM	Prosperini et al. (2012)
Caco-2	Intestine	3.1–25 µM 24 h, 48 h, 72 h	MTT	24 h: 20.6 µM 48 h: 12.8 µM 72 h: 3.2 µM	Prosperini et al. (2013a)
Caco-2	Intestine	3.1–25 µM 24 h, 48 h, 72 h	NR	24 h: 8.8 µM 48 h: 3.4 µM 72 h: 1.9 µM	Prosperini et al. (2013a)
Caco-2	Intestine	0–100 µM 48h	Resazurin-based toxicity assay	3.9 µM	Olleik et al. (2019)
CCRF-CEM	Blood	1–10 μM 24 h	MTT	2.5 μM	Jow et al. (2004)
CFU-GM	Blood	6.4 nM to 64 µM 12–14 days	Clonogenic assay	3.4 µM	Ficheux et al. (2012)
CFU-MK	Blood	6.4 nM to 64 µM 12–14 days	Clonogenic assay	0.7 µM	Ficheux et al. (2012)
CHO-K1	Ovary	0.78 to 12.5 µM 24 h, 48 h, 72 h	NR	24 h: 17.2 µM 48 h: 6.2 µM 72 h: 3.8 µM	Ruiz et al. (2011a)
CHO-K1	Ovary	0.78–12.5 µM 24 h, 48 h, 72 h	MTT	Values similar to NR (data not shown)	Ruiz et al. (2011a)
CHO-K1	Ovary	0.75–20 µM 24h, 48h, 72h	MTT	24h: 10.7 µM 48h: 2.5 µM 72h: 2.2 µM	Zouaoui et al. (2016)
CHO-K1	Ovary	2.5–20 µM 24h, 48h, 72h	MTT	24h: 10.7 µM 48h: 2.5 µM 72h: 2.2 μM	Mallebrera et al. (2016)
Dendritic cells (human): immature mature	Blood	n.m 3 h	MTS	1.0 µM 2.9 µM	Ficheux et al. (2013)
H4IIE	Liver	0–25 µM 4h, 24h	MTT	4h: 3.0 µM 24h: 1.9 µM	Watjen et al. (2014)
HCT15	Intestine	0–10 µM 72 h	SRB	1.9 µM	Lee et al. (2008)
HCT116	Intestine	0–25 µM 24h	MTT	2.4 µM	Watjen et al. (2014)
HEK	Skin	0–100 µM 48h	Resazurin-based toxicity assay	5.4 µM	Olleik et al. (2019)
HepaRG (differentiated)	Liver	8.5 nM to 34 µM 48 h	CellTiter 96	4.5 µM	Coulet et al. (2024)
HepG2	Liver	16 nM to 510 µM 24 h	AB	8.8—22.5 µM	Ivanova et al. (2006)
HepG2	Liver	16 nM to 510 µM 24 h	BrdU	1.4—4.0 µM	Ivanova et al. (2006)
HepG2	Liver	0–25 µM 24h, 48h, 72h	MTT	24h: 12.5 µM 48h: 7.0 µM 72h: 5.5 µM	Juan-Garcia et al. (2019)
HepG2	Liver	0–25 µM 24h	MTT	3.6 µM	Watjen et al. (2014)
HepG2	Liver	0.05–5 µM 24h, 48h	MTT	24h: 1.2 µM 48h: 1.0 µM	Juan-Garcia et al. (2021a)
HepG2	Liver	0–100 µM 48h	Resazurin-based toxicity assay	3.4 µM	Olleik et al. (2019)
HepG2	Liver	0.2–30 μM 24 h	MTT SRB NR	~ 60% viable cells at 2.5 µM * ~ 50% viable cells at 2.5 µM * ~ 60% viable cells at 10 µM *	Ornelis et al. (2019)
HL-60	Blood	0–16 µM 4 h, 24 h	Flow cytometry with DAPI	4h: n.r 24h: 4 µM	Juan-Garcia et al. (2024)
HL-60 (undifferentiated and differentiated)	Blood	100 nM to 300 μM 4 h, 24 h	TB	4h: n.m 24h: 15 µM (undifferentiated) 20 µM (differentiated)	Calo et al. (2004)
HT-29	Intestine	0.6–30 µM 24 h, 48 h	MTT	24 h: 15.0 µM 48 h: 9.7 µM	Prosperini et al. (2012)
HUVEC	Umbilical cord	0–100 µM 48h	Resazurin-based toxicity assay	2.4 µM	Olleik et al. (2019)
IPEC-1	Intestine	n.m 48h	CellTiter-Glo®	4.3 µM	Khoshal et al. (2019)
IPEC-J2	Intestine	0–20 µM 48 h	SRB	2.4 µM	Novak et al. (2019)
IPEC-J2	Intestine	5–100 μM 24 h	Annexin V-FITC	Complete cell disruption at 10 µM	Fraeyman et al. (2018b)
J774	Ascites	0–150 μM 24 h	TB	11.0 μM	Tomoda et al. (1992)
Jurkat-T	Blood	1–15 µM 24 h, 48 h, 72 h	MTT	24 h: 7.5 μM 48 h: 5.0 μM 72 h: 3.0 μM	Manyes et al. (2018)
Macrophages (human)	Blood	n.m 3 h	MTS	2.5 µM	Ficheux et al. (2013)
MDA-MB-231	Breast	0–16 µM 4 h, 24 h	Flow cytometry with DAPI	4h: 3.75 µM 24h: 3 µM	Juan-Garcia et al. (2024)
MRC-5	Lung	16 nM to 510 µM 24 h	AB	4.7—5.0 µM	Ivanova et al. (2006)
MRC-5	Lung	16 nM to 510 µM 24 h	BrdU	0.6—1.1 µM	Ivanova et al. (2006)
N87	Stomach	0–100 µM 48h	Resazurin-based toxicity assay	27.5 µM	Olleik et al. (2019)
PBMC	Blood	0–16 µM 4 h, 24 h	Flow cytometry with DAPI	4h: 0.06 µM 24h: 0.5 µM	Juan-Garcia et al. (2024)
PK15	Kidney	0–15 µM 24 h	MTT	5 μM	Klaric et al. (2010)
Primary hepatocytes (*Salmo salar*)	Liver	0–10 μM 48 h	XCELLigence	5.0 µM; impedance reduced by 29% at 10 μM	Søderstrøm et al. (2022)
Primary hepatocytes (*Salmo salar*)	Liver	0–10 μM 48 h	MTT	2.6 µM	Søderstrøm et al. (2022)
Primary hepatocytes (*Salmo salar*)	Liver	0–10 μM 48 h	NR	2.6 µM	Søderstrøm et al. (2022)
Primary hepatocytes (*Salmo salar*)	Liver	0–5 μM 48 h	ATP	Decreased by ≥ 80% at 5 µM	Søderstrøm et al. (2022)
Primary hepatocytes (*Salmo salar*)	Liver	0–5 μM 48 h	H_2_O_2_	Reduced by ≥ 70% at 5 μM	Søderstrøm et al. (2022)
SF-9	Ovary	10 μM to 10 mM 48h	MTT	2.5 µM	Fornelli et al. (2004)
SF-9	Ovary	10 μM to 10 mM 48h	TP	3 µM	Fornelli et al. (2004)
SH-SY5Y	Bone	0.04–2.5 µM 24h, 48h	MTT	24h: no effect on viability 48h: 43% viable cells at 2.5 µM	Agahi et al. (2022)
SK-MEL-2	Skin	0–10 µM 72 h	SRB	1.5 µM	Lee et al. (2008)
SK-OV-3	Ovary	0–10 µM 72 h	SRB	1.4 µM	Lee et al. (2008)
U397	Blood	100 nM to 300 μM 4 h, 24 h	TB	4h: n.m 24h: 30 µM	Calo et al. (2004)
V79	Lung	0–10 mM 48 h	NR	1.6 µM	Behm et al. (2012)
Vero	Kidney	0.78–25 µM 24 h, 48 h, 72 h	NR	6.25—10.02 µM	Ruiz et al. (2011b)
Vero	Kidney	0.78–25 µM 24 h, 48 h, 72 h	MTT	6.77—11.08 µM	Ruiz et al. (2011b)
Enniatin A					
BEAS-2B	Lung	0–100 µM 48h	Resazurin-based toxicity assay	5.7 µM	Olleik et al. (2019)
Caco-2	Intestine	0.9–15 µM 24 h, 48 h, 72 h	MTT	24 h: n.r.; concentration-dependent effect 48 h: 6.8 μM 72 h: 1.6 μM	Prosperini et al. (2014)
Caco-2	Intestine	0.6–30 µM 24 h, 48 h	MTT	24 h: n.r.; concentration-dependent effect 48 h: 9.3 µM	Meca et al. (2011a)
Caco-2	Intestine	0–100 µM 48h	Resazurin-based toxicity assay	1.1 µM	Olleik et al. (2019)
CHO-K1	Ovary	0.46–7.5 µM 24 h, 48 h, 72 h	MTT	24 h: n.r.; concentration-dependent effect 48 h: 2.8 µM 72 h: 3.3 µM	Lu et al. (2013)
CHO-K1	Ovary	0–15 μM 24 h	MTT	n.r.; concentration-dependent effect	Lombardi et al. (2012)
HEK	Skin	0–100 µM 48h	Resazurin-based toxicity assay	2.0 µM	Olleik et al. (2019)
HeLa	Uterus	0.07–9.17 µM 24 h, 48 h	MTT	24 h: 0.7 µM 48 h: 0.4 µM	Mamur et al. (2018)
HepaRG (differentiated)	Liver	0.97 nM to 39 µM 48 h	CellTiter 96	10.9 µM	Coulet et al. (2024)
HepG2	Liver	0.6 to 30 µM 24 h, 48 h	MTT	24 h: 26.2 µM 48 h: 11.4 µM	Meca et al. (2011a)
HepG2	Liver	0–100 µM 48h	Resazurin-based toxicity assay	3.0 µM	Olleik et al. (2019)
HepG2	Liver	0.04 nM to 82.2 µM 24 h	AB	7.3- 9.4 µM	Ficheux et al. (2013)
HepG2	Liver	0.04 nM to 82.2 µM 24 h	BrdU	1.6—2.5 µM	Ivanova et al. (2006)
HT-29	Intestine	0.6 to 30 µM 24 h, 48 h	MTT	24 h: n.r.; concentration-dependent effect 48 h: 8.2 µM	Meca et al. (2011a)
HUVEC	Umbilical cord	0–100 µM 48h	Resazurin-based toxicity assay	2.8 µM	Olleik et al. (2019)
IPEC-J2	Intestine	0–20 µM 48 h	SRB	3.4 µM	Novak et al. (2019)
IPEC-J2	Intestine	5–100 μM 24 h	Annexin V-FITC	32% of viable cells at 10 µM	Fraeyman et al. (2018b)
J774	Ascites	0–150 μM 24 h	TB	2.6 μM	Tomoda et al. (1992)
MRC-5	Lung	0.04 nM to 82.2 µM 24 h	AB	3.5—3.8 µM	Ivanova et al. (2006)
MRC-5	Lung	0.04 nM to 82.2 µM 24 h	BrdU	0.6—0.8 µM	Ivanova et al. (2006)
N87	Stomach	0–100 µM 48h	Resazurin-based toxicity assay	0.01 µM	Olleik et al. (2019)
Enniatin A1					
BEAS-2B	Lung	0–100 µM 48h	Resazurin-based toxicity assay	6.4 µM	Olleik et al. (2019)
C6	Brain	0–25 µM 24 h	MTT	2.5—10 µM	Watjen et al. (2009)
Caco-2	Intestine	0.9–15.0 µM 24 h, 48 h, 72 h	MTT	24 h: 14.8 μM 48 h: 7.7 μM 72 h: 1.3 μM	Prosperini et al. (2014)
Caco-2	Intestine	0–100 µM 48h	Resazurin-based toxicity assay	2.7 µM	Olleik et al. (2019)
Caco-2	Intestine	0.6–30 µM 24 h, 48 h	MTT	24 h: 12.3 µM 48 h: 2.7 µM	Meca et al. (2011a)
Caco-2 (undifferentiated)	Intestine	0–30 µM 24 h	MTT	12.3 µM	Meca et al. (2010)
CHO-K1	Ovary	0.46–15 µM 24 h, 48 h, 72 h	MTT	24 h: 8.8 µM 48 h: 1.7 µM 72 h: 1.7 µM	Lu et al. (2013)
CHO-K1	Ovary	0–15 μM 24 h	MTT	8.8 µM	Lombardi et al. (2012)
H4IIE	Liver	0–25 µM 24 h	MTT	1—1.5 µM	Watjen et al. (2009)
HEK	Skin	0–100 µM 48h	Resazurin-based toxicity assay	2.3 µM	Olleik et al. (2019)
HepaRG (differentiated)	Liver	0.25 nM to 10 µM 48 h	CellTiter 96	3.36 µM	Coulet et al. (2024)
HepG2	Liver	0.6–30 µM 24 h, 48 h	MTT	24 h: 11.6 µM 48 h: 2.6 µM	Meca et al. (2011a)
HepG2	Liver	0–25 µM 24 h	MTT	10 µM	Watjen et al. (2009)
HepG2	Liver	0.08 nM to 159 µM 24 h	AB	11.7—18.1 µM	Ivanova et al. (2006)
HepG2	Liver	0.08 nM to 159 µM 24 h	BrdU	2.6—4.2 µM	Ivanova et al. (2006)
HepG2	Liver	0–100 µM 48h	Resazurin-based toxicity assay	5.6 µM	Olleik et al. (2019)
HT-29	Intestine	0.6–30 µM 24 h, 48 h	MTT	24 h: 9.1 µM 48 h: 1.4 µM	Meca et al. (2011a)
HUVEC	Umbilical cord	0–100 µM 48h	Resazurin-based toxicity assay	4.6 µM	Olleik et al. (2019)
IPEC-J2	Intestine	0–20 µM 48 h	SRB	4.2 µM	Novak et al. (2019)
IPEC-J2	Intestine	5–100 μM 24 h	Annexin V-FITC	87% of viable cells at 10 µM	Fraeyman et al. (2018b)
J774	Ascites	0–150 μM 24 h	TB	2.6 μM	Tomoda et al. (1992)
MRC-5	Lung	0.08 nM to 159 µM 24 h	AB	5.9—6.9 µM	Ivanova et al. (2006)
MRC-5	Lung	0.08 nM to 159 µM 24 h	BrdU	1.1—1.4 µM	Ivanova et al. (2006)
N87	Stomach	0–100 µM 48h	Resazurin-based toxicity assay	0.003 µM	Olleik et al. (2019)
SH-SY5Y	Bone	0.1–15 μM 6 h, 24 h	MTT	6 h: 3.4 μM 24 h: 2.0 μM	Perez-Fuentes et al. (2022)
SH-SY5Y	Bone	0.1–15 μM 6 h, 24 h	LDH	6 h: ~ 58% of increased release > 10 μM 24 h: 6.2 μM	Perez-Fuentes et al. (2022)
Vero	Kidney	3–192 μM (2–128 µg/mL) 24 h	WST-1	> 64 µg/mL (43 µM)	Wang et al. (2019)
Enniatin A2					
Caco-2	Intestine	0.6–30 µM 24 h, 48 h	MTT	24 h: 18.7 µM 48 h: 2.6 µM	Meca et al. (2011a)
HepG2	Liver	0.6–30 µM 24 h, 48 h	MTT	n.r.; concentration-dependent effect	Meca et al. (2011a)
HT-29	Liver	0.6–30 µM 24 h, 48 h	MTT	n.r.; concentration-dependent effect	Meca et al. (2011a)
Enniatin B					
Balb 3T3	Embryo	1.5–100 µM 24 h	AK release	No effect	Jonsson et al. (2016)
Balb 3T3	Embryo	1.5–100 µM 24 h	ATP levels	8.4 µM	Jonsson et al. (2016)
Balb 3T3	Embryo	3–12 µM 24 h	BrdU	4.2 µM	Jonsson et al. (2016)
Balb 3T3	Embryo	11–45 µM 24 h, 48 h	Annexin V-FITC	24 h: 86—84% viable cells; 1—4% early apoptotic cells; IC_50_ ~ 11 µM Similar findings at 48 h	Jonsson et al. (2016)
BEAS-2B	Lung	0–100 µM 48h	Resazurin-based toxicity assay	43.7 µM	Olleik et al. (2019)
Caco-2	Intestine	1.5–4 µM 72h	MTT	2.1 µM	de Sa et al. (2024)
Caco-2	Intestine	1–10 µM 24h	WST-1	6.3 µM	Vejdovszky et al. (2016)
Caco-2	Intestine	0.31–10 µM 24h, 48h, 72h	MTT	24h: n.r.; concentration-dependent effect 48h: n.r.; concentration-dependent effect 72h: 3.9 µM	Fernandez-Blanco et al. (2016a)
Caco-2	Intestine	0.9–15.0 µM 24 h, 48 h, 72 h	MTT	24 h: n.r.; concentration-dependent effect 48 h: n.r.; concentration-dependent effect 72 h: 11.7 μM	Prosperini et al. (2014)
Caco-2	Intestine	0–100 µM 48h	Resazurin-based toxicity assay	4.6 µM	Olleik et al. (2019)
Caco-2	Intestine	0.6–30 µM 24 h, 48 h	MTT	24 h, 48 h: n.r	Meca et al. (2011a)
Caco-2 (undifferentiated and differentiated)	Intestine	0.6–30 μM 24 h, 48 h	MTT	24 h, 48 h: n.r	Meca et al. (2011a)
CCF-STTG1	Brain	0.1–10 μM 48 h	CCK-8	8.9 µM	Krug et al. (2018)
CCF-STTG1	Brain	0.1–2.5 μM 48 h	Caspase-3 activation	2.7-fold increase at 2.5 μM	Krug et al. (2018)
CCF-STTG1	Brain	0.1–2.5 μM 48 h	LDH	No effect	Krug et al. (2018)
CHO-K1	Ovary	0.46–15 µM 24 h, 48 h, 72 h	MTT	24 h: 11 µM 48 h: 2.4 µM 72 h: 2.8 µM	Lu et al. (2013)
CHO-K1	Ovary	0–15 μM 24 h	MTT	11 µM	Lombardi et al. (2012)
CIEB clone 9	Intestine	0–200 µM 48 h	WST-1	6.7 µM	Reisinger et al. (2019)
CIEB clone 9	Intestine	0–200 µM 48 h	NRU	4 µM	Reisinger et al. (2019)
CIEB clone 9	Intestine	0–200 µM 48 h	SRB	n.r	Reisinger et al. (2019)
Dendritic cells (human): immature mature	Blood	0–10 µM 3 h	MTS	1.6 µM 2.6 µM	Ficheux et al. (2013)
H295R	Adrenal gland	0.01–100 µM 48 h	AB	37% of viable cells at 100 µM	Kalayou et al. (2015)
HBMEC	Brain	0.1–10 μM 48 h	CCK-8	No effect	Krug et al. (2018)
HEK	Skin	0–100 µM 48h	Resazurin-based toxicity assay	54.2 µM	Olleik et al. (2019)
HEK293	Kidney	1.59–5.92 µM 72 h	MTT	1.2 µM	de Sa et al. (2024)
HepaRG (differentiated)	Liver	1 nM to 41.8 µM 48 h	CellTiter 96	11.9 µM	Coulet et al. (2024)
HepG2	Liver	0.67–2 µM 72 h	MTT	4 µM	de Sa et al. (2024)
HepG2	Liver	1.5–100 µM 24 h	AK release	No effect	Jonsson et al. (2016)
HepG2	Liver	1.5–100 µM 24 h	ATP levels	2.9 µM	Jonsson et al. (2016)
HepG2	Liver	3–12 µM 24 h	BrdU	0.5 µM	Jonsson et al. (2016)
HepG2	Liver	0.5–30 µM 24 h, 48 h	MTT	24 h: n.r.; concentration-dependent effect 48 h: n.r.; concentration-dependent effect	Meca et al. (2011a)
HepG2	Liver	0–100 µM 48h	Resazurin-based toxicity assay	3.4 µM	Olleik et al. (2019)
HepG2	Liver	3 nM to 2372 µM 24 h	AB	206.7—435.9 μM	Ivanova et al. (2006)
HepG2	Liver	3 nM to 2372 µM 24 h	BrdU	0.9—1.1 μM	Ivanova et al. (2006)
HL-60	Blood	0–8 µM 4 h, 24 h	Flow cytometry with DAPI	4h: 0.5 µM 24h: 0.25 µM	Juan-Garcia et al. (2024)
HT-29	Intestine	0.5–30 µM 24 h, 48 h	MTT	24 h: n.r.; concentration-dependent effect 48 h: 2.8 µM	Meca et al. (2011a)
HUVEC	Umbilical cord	0–100 µM 48h	Resazurin-based toxicity assay	17.3 µM	Olleik et al. (2019)
IPEC-1	Intestine	n.m 48h	CellTiter-Glo®	4.4 µM	Khoshal et al. (2019)
IPEC-J2	Intestine	0–20 µM 48 h	SRB	3.3 µM	Novak et al. (2019)
IPEC-J2	Intestine	5–100 μM 24 h	Annexin V-FITC	83% of viable cells at 100 μM	Fraeyman et al. (2018b)
J774	Ascites	0–150 μM 24 h	TB	> 10 μM	Tomoda et al. (1992)
Jurkat-T	Blood	1–15 µM 24 h, 48 h, 72 h	MTT	Decreased cell viability at 15 µM: 21% (24 h), 23% (48 h), 29% (72 h)	Manyes et al. (2018)
LC unstimulated stimulated	Testis	0.01–100 µM 48 h	AB	80% viable cells at 100 µM 79% viable cells at 100 µM	Kalayou et al. (2015)
Macrophages (human)	Blood	0–10 µM 3 h	MTS	2.5 µM	Ficheux et al. (2013)
MDA-MB-231	Breast	0–8 µM 4 h, 24 h	Flow cytometry with DAPI	4h: n.r 24h: 0.15 µM	Juan-Garcia et al. (2024)
MRC-5	Lung	3 nM to 2372 µM 24 h	AB	1.9—9.8 μM	Ivanova et al. (2006)
MRC-5	Lung	3 nM to 2372 µM 24 h	BrdU	1.9—3.6 μM	Ivanova et al. (2006)
N87	Stomach	0–100 µM 48h	Resazurin-based toxicity assay	1.7 µM	Olleik et al. (2019)
N87	Stomach	1.23–5.92 µM 72h	MTT	3.3 µM	de Sa et al. (2024)
vPBCEC	Brain	0.1–10 μM 48 h	CCK-8	70% viable cells > 5 µM	Krug et al. (2018)
PBMC	Blood	0–16 µM 4 h, 24 h	Flow cytometry with DAPI	4h: 0.03 µM 24h: 5 µM	Juan-Garcia et al. (2024)
Primary hepatocytes (*Salmo salar*)	Liver	0–10 μM 48 h	XCELLigence	3.2 µM; impedance reduced by 34% at 10 μM	Søderstrøm et al. (2022)
Primary hepatocytes (*Salmo salar*)	Liver	0–10 μM 48 h	MTT	5.7 µM	Søderstrøm et al. (2022)
Primary hepatocytes (*Salmo salar*)	Liver	0–10 μM 48 h	NR	11.1 µM	Søderstrøm et al. (2022)
Primary hepatocytes (*Salmo salar*)	Liver	0–5 μM 48 h	ATP	Decreased by ≥ 80% at 5 µM	Søderstrøm et al. (2022)
Primary hepatocytes (*Salmo salar*)	Liver	0–5 μM 48 h	H_2_O_2_	Reduced by ≥ 70% at 5 μM	Søderstrøm et al. (2022)
RAW264.7	Ascites	0.05–100 µM 24 h	NR	2.6 µM	Gammelsrud et al. (2012)
RAW264.7	Ascites	0.05–100 µM 24 h	AB	4.7 µM	Gammelsrud et al. (2012)
V79	Lung	0–100 µM 48 h	NR	4 µM	Behm et al. (2012)
V79	Lung	0.1–75 µM 24 h, 48 h	NR	24 h: 36 µM 48 h: 4 µM	Follmann et al. (2009)
V79	Lung	0.1–75 µM 24 h, 48 h	AB	24 h: 34 µM 48 h: 2.5 µM	Follmann et al. (2009)
V79	Lung	0.1–75 µM 24 h, 48 h	BCA	24 h: 43 µM 48 h: 3.9 µM	Follmann et al. (2009)
V79	Lung	0–70 µM 24 h, 48 h	NR	24 h: 36 μM 48 h: 4 μM	Behm et al. (2009)
Enniatin B1					
BEAS-2B	Lung	0–100 µM 48h	Resazurin-based toxicity assay	12.7 µM	Olleik et al. (2019)
Caco-2	Intestine	0.9–15 µM 24 h, 48 h, 72 h	MTT	24 h: n.r.; concentration-dependent effect 48 h: 11.3 µM 72 h: 2.8 µM	Prosperini et al. (2014)
Caco-2	Intestine	1–25 µM 3 h, 24 h	NR	3 h: 10 μM 24 h: 2.1 μM	Ivanova et al. (2012)
Caco-2	Intestine	4.9 pM to 482.5 µM 24 h	AB	68.6 µM	Ivanova et al. (2010)
Caco-2	Intestine	4.9 pM to 482.5 µM 24 h	NR	9.9 µM	Ivanova et al. (2010)
Caco-2	Intestine	4.9 pM to 482.5 µM 24 h	LDH	20.6 µM	Ivanova et al. (2010)
Caco-2	Intestine	0–100 µM 48h	Resazurin-based toxicity assay	3.1 µM	Olleik et al. (2019)
Caco-2	Intestine	0–30 µM 24 h, 48 h	MTT	24 h: 19.5 μM 48 h: 11.5 μM	Meca et al. (2011a)
CCF-STTG1	Brain	0.1–10 μM 48hh	CCK-8	4.4 µM	Krug et al. (2018)
CCF-STTG1	Brain	0.1–2.5 μM 48 h	Caspase-3 activation	Increase to 160% at 1 µM	Krug et al. (2018)
CCF-STTG1	Brain	0.1–2.5 μM 48 h	LDH	No effect	Krug et al. (2018)
CHO-K1	Ovary	0.46–15 µM 24 h, 48 h, 72 h	MTT	24 h: 4.5 μM 48 h: 2.6 μM 72 h: 2.5 μM	Lu et al. (2013)
CHO-K1	Ovary	0–15 μM 24 h	MTT	4.5 μM	Lombardi et al. (2012)
HBMEC	Brain	0.1–10 μM 48 h	CCK-8	No effect	Krug et al. (2018)
HEK	Skin	0–100 µM 48h	Resazurin-based toxicity assay	3.3 µM	Olleik et al. (2019)
HepaRG (differentiated)	Liver	1 nM to 40.8 µM 48 h	CellTiter 96	8.6 µM	Coulet et al. (2024)
HepG2	Liver	0–30 µM 24 h, 48 h	MTT	24 h: 24.3 μM 48 h: 8.5 μM	Meca et al. (2011a)
HepG2	Liver	2 nM to 1182 µM 24 h	AB	9.2—36 µM	Ivanova et al. (2006)
HepG2	Liver	2 nM to 1182 µM 24 h	BrdU	2.8—3.5 µM	Ivanova et al. (2006)
HepG2	Liver	0–100 µM 48h	Resazurin-based toxicity assay	5.6 µM	Olleik et al. (2019)
HT-29	Intestine	0–30 µM 24 h, 48 h	MTT	24 h: 16.8 μM 48 h: 3.6 μM	Meca et al. (2011a)
HUVEC	Umbilical cord	0–100 µM 48h	Resazurin-based toxicity assay	7.0 µM	Olleik et al. (2019)
IPEC-1	Intestine	0.3–100 µM 48h	CellTiter-Glo®	15.8 µM	Kolf-Clauw et al. (2013)
IPEC-1	Intestine	n.m 48h	CellTiter-Glo®	13.5 µM	Khoshal et al. (2019)
IPEC-J2	Intestine	0–20 µM 48 h	SRB	3.7 µM	Novak et al. (2019)
IPEC-J2	Intestine	5–100 μM 24 h	Annexin V-FITC	93% of viable cells at 10 µM	Fraeyman et al. (2018b)
J774	Ascites	0–150 μM 24 h	TB	> 5 μM	Tomoda et al. (1992)
MDCK-II -Wild-type -MDR1 -MRP2 -BCRP	Kidney	4.9–482.5 µM 24 h	AB	2.2 µM 6.5 µM 8.8 µM 6.1 µM	Ivanova et al. (2010)
MDCK-II -Wild-type -MDR1 -MRP2 -BCRP	Kidney	4.9–482.5 µM 24 h	NR	27 µM 29 µM 32 µM 30 µM	Ivanova et al. (2010)
MDCK-II -Wild-type -MDR1 -MRP2 -BCRP	Kidney	4.9–482.5 µM 24 h	LDH	16 µM 19 µM 21 µM 19 µM	Ivanova et al. (2010)
MEF	Embryo	0.6–40 µM 24 h	NR	1.5—1.7 µM	Oliveira et al. (2020)
Mouse embryos (blastocyst-stage)	Embryo	1–10 μM 24 h	TUNEL	Increase in apoptotic cells at 5 and 10 μM	Huang et al. (2019)
MRC-5	Lung	0.25 nM to 482 µM 24 h	AB	6.1 µM	Ivanova and Uhlig (2008)
MRC-5	Lung	0.25 nM to 482 µM 24 h	NR	10 µM	Ivanova and Uhlig (2008)
MRC-5	Lung	0.25 nM to 482 µM 24 h	LDH	21 µM	Ivanova and Uhlig (2008)
MRC-5	Lung	2 nM to 1182 µM 24 h	AB	4.5—4.7 µM	Ivanova et al. (2006)
MRC-5	Lung	2 nM to 1182 µM 24 h	BrdU	1.2—1.4 µM	Ivanova et al. (2006)
N87	Stomach	0–100 µM 48h	Resazurin-based toxicity assay	0.008 µM	Olleik et al. (2019)
PBCEC	Brain	0.1–10 μM 48 h	CCK-8	64% viable cells at 10 µM	Krug et al. (2018)
SH-SY5Y	Bone	0.1–15 μM 6 h, 24 h	MTT	6 h: 3.0 μM 24 h: 2.7 µM	Perez-Fuentes et al. (2022)
SH-SY5Y	Bone	0.1–15 μM 6 h, 24 h	LDH	6 h: 7.4 μM 24 h: 6.7 μM	Perez-Fuentes et al. (2022)
THP-1	Blood	0–5 µM 24 h	AB	IC_20_ = 1.2 µM	Solhaug et al. (2016)
Enniatin B2					
HepG2	Liver	0.1 nM to 51.2 µM 24 h	BrdU	4.0—12.9 μM	Ivanova et al. (2006)
Enniatin B3					
HepG2	Liver	0.05 nM to 19.6 µM 24 h	BrdU	3.4—14.4 μM	Ivanova et al. (2006)
Enniatin B4					
Caco-2	Intestine	0.5–30 µM 24 h, 48 h	MTT	24 h: 4.5 µM 48 h: 20.6 µM	Meca et al. (2011a)
HepG2	Liver	0.5–30 µM 24 h, 48 h	MTT	24 h: 8.5 µM 48 h: 24.3 µM	Meca et al. (2011a)
HT-29	Intestine	0.5–30 µM 24 h, 48 h	MTT	24 h: 3.7 µM 48 h: 16.8 µM	Meca et al. (2011a)
Enniatin H					
A549	Lung	0–10 µM 72 h	SRB	1.8 µM	Lee et al. (2008)
HCT15	Intestine	0–10 µM 72 h	SRB	2.5 µM	Lee et al. (2008)
HCT15	Intestine	0.1–30 µM 72 h	SRB	16.7 µM	Hwang and Lee (2024)
HCT15/CL05	Intestine	0.1–30 µM 72 h	SRB	17.7 µM	Hwang and Lee (2024)
MES-SA	Uterus	0.1–30 µM 72 h	SRB	12.9 µM	Hwang and Lee (2024)
MES-SA/DX5	Uterus	0.1–30 µM 72 h	SRB	14.9 µM	Hwang and Lee (2024)
SK-MEL-2	Skin	0–10 µM 72 h	SRB	1.8 µM	Lee et al. (2008)
SK-OV-3	Ovary	0–10 µM 72 h	SRB	1.7 µM	Lee et al. (2008)
Enniatin I					
A549	Lung	0–10 µM 72 h	SRB	0.5 µM	Lee et al. (2008)
HCT15	Intestine	0–10 µM 72 h	SRB	0.5 µM	Lee et al. (2008)
HCT15	Intestine	0.1–30 µM 72 h	SRB	5.5 µM	Hwang and Lee (2024)
HCT15/CL05	Intestine	0.1–30 µM 72 h	SRB	5.8 µM	Hwang and Lee (2024)
MES-SA	Uterus	0.1–30 µM 72 h	SRB	3.9 µM	Hwang and Lee (2024)
MES-SA/DX5	Uterus	0.1–30 µM 72 h	SRB	4.9 µM	Hwang and Lee (2024)
SK-MEL-2	Skin	0–10 µM 72 h	SRB	0.5 µM	Lee et al. (2008)
SK-OV-3	Ovary	0–10 µM 72 h	SRB	0.5 µM	Lee et al. (2008)
Enniatin J3					
Caco-2 (differentiated and undifferentiated)	Intestine	0.6–30 µM 24 h, 48 h	MTT	n.r.; concentration-dependent effect	Meca et al. (2011b)
Caco-2	Intestine	0.6–30 µM 24 h, 48 h	MTT	n.r.; concentration-dependent effect	Meca et al. (2011a)
HepG2	Liver	0.6–30 µM 24 h, 48 h	MTT	n.r.; concentration-dependent effect	Meca et al. (2011a)
HT-29	Intestine	0.6–30 µM 24 h, 48 h	MTT	n.r.; concentration-dependent effect	Meca et al. (2011a)
Enniatin MK1688					
HCT15	Intestine	0.1–30 µM 72 h	SRB	14 µM	Hwang and Lee (2024)
HCT15/CL05	Intestine	0.1–30 µM 72 h	SRB	16.3 µM	Hwang and Lee (2024)
MES-SA	Uterus	0.1–30 µM 72 h	SRB	13.6 µM	Hwang and Lee (2024)
MES-SA/DX5	Uterus	0.1–30 µM 72 h	SRB	13.6 µM	Hwang and Lee (2024)

*A375SM* human melanoma, *A549* human lung adenocarcinoma, *Balb* 3T3 mouse fibroblasts, *BEAS-2B* human normal lung epithelial, *BC-1* human breast cancer, *BFU-E* human red blood progenitor cells, *C6* rat glioma, *Caco-2* human colon adenocarcinoma, *CCF-STTG1* human astrocytoma, *CCRF-CEM* human leukemic lymphoblasts, CFU-*GM* human white blood progenitor cells, *CFU-MK* human platelet progenitor cells, *CIEB* clone 9 calf small intestinal epithelial, *CHO-K1* Chinese hamster ovary cells, *H295R* human adrenocortical carcinoma, *H4IIE* rat hepatoma, *HBMEC* human brain microvascular endothelial, *HCT15* human colorectal adenocarcinoma, *HCT15/CL05* multidrug resistant subline of HCT15, *HCT116* human colon carcinoma, *HEK* human normal epidermal keratinocytes, *HEK293* human embryonal kidney, *HeLa* human cervix carcinoma, human acute myeloid leukaemia, *HepaRG* human hepatoma, HepG2 human hepatocarcinoma, *HL-60* human promyelocytic leukaemia, *HT-29* human colorectal adenocarcinoma, *HUVEC* Human Umbilical Vein Endothelial Cells, *IPEC-1* intestinal porcine epithelial, *IPEC-J2* intestinal porcine epithelial jejunal, *J774* murine macrophages, *Jurkat-T* human leukemic lymphoblasts, *KB* human epidermoid carcinoma, *LC* neonatal porcine Leydig cells, *MDA-MB-231* human breast cancer, *MDCK-II* canine kidney epithelial cells, *MDCKII-MDR1* canine kidney epithelial cells overexpressing human P-gp, *MDCKII-MRP2* canine kidney epithelial cells overexpressing human multidrug resistance-associated protein 2, *MDCKII-BCRP* canine kidney epithelial cells overexpressing human breast cancer resistance protein, *MEF* mouse embryonic fibroblasts, *MES-SA* human uterine sarcoma, *MES-SA/DX5* multidrug resistant subline of MES-SA, *MRC-5* human foetal lung fibroblasts, *N87* human gastric cell line, *PBCEC* porcine brain capillary endothelial, *PBMC* human primary peripheral blood mononuclear cells, *PK15* porcine kidney epithelial, *RAW264*.*7* murine macrophages, *SF-9* lepidopteran (Spodoptera frugiperda), *SH-SY5Y* human neuroblastoma, *SK-MEL-2* human melanoma, *SK-OV-3* human ovarian adenocarcinoma, *SK-MEL-2* human melanoma, *THP-1* human leukaemia monocytic cells, *U397* human monocytic lymphoma, *V79* Chinese hamster lung fibroblasts, *Vero* monkey kidney epithelial, *AB* Alamar Blue, *Annexin V-FITC* annexin V conjugated with fluorescein isothiocyanate, *AK* Adenylate Kinase, *ATP* Adenosine triphosphate, *BrdU* Bromodeoxyuridine, *CCK-8* Cell Counting kit-8, *DAPI* 4′,6-diamidino-2-phenylindole, *IC*_*20*_ 20% inhibitory concentration, *LDH* Lactate Dehydrogenase, *MTS* 3-(4,5-dimethylthiazol-2-yl)-5-(3-carboxymethoxyphenyl)-2-(4-sulfophenyl)-2H-tetrazolium, *MTT* 3-(4,5-dimethylthiazol-2-yl)-2,5-diphenyltetrazolium bromide, *NR* Neutral Red, *SRB* Sulforhodamine B, *TUNEL* Terminal deoxynucleotidyl transferase dUTP nick end labelling, *TB* Trypan Blue, *WST* 2-(4-iodophenyl)-3-(4-nitrophenyl)-5-(2,4-disulfophenyl)-2H-tetrazolium, *n.m.* not mentioned, *n.r.* not reached

*Read from chart by the authors of this study

Besides conventional cytotoxicity testing, some studies have used transcriptomics to explore and understand the mechanisms mediating the toxicity of these mycotoxins, particularly in liver and immune system cells. Using genome-wide expression analysis with rat primary hepatocytes exposed to 1, 10, and 20 μM ENN B for 1 h and 4 h, Jonsson et al. (2016) showed that ENN B modifies mitochondrial organisation and induces dysfunction of the mitochondrial electron transfer chain, leading to alterations in energy metabolism. In a more recent work using liver HepaRG spheroids, several affected pathways were identified following cells exposure to ENN B and ENN B1, including complement cascades, metabolism, steroid hormone biosynthesis, bile secretion, and cholesterol pathways (Coulet et al. 2024). Additional knowledge was provided by toxicogenomic studies that revealed changes in the pattern of gene expression in human Jurkat lymphoblastic T-cells exposed to ENN B (1.5, 3 and 5 μM, 24 h), BEA (1.5, 3 and 5 μM, 24 h) and a mixture (1:1) of both mycotoxins (BEA and ENN B, 0.1, 0.5, 1.5 μM, 24 h) (Alonso-Garrido et al. 2018; Escriva et al. 2019, 2018). Based on the analysis of genes differentially expressed (RNA sequencing and bioinformatic analysis) in exposed compared to control cells, the authors concluded that the biological processes, molecular functions and pathways mainly affected by ENN B-treatment were related to mitochondrial metabolism and cellular respiration and, thereby, mitochondria were the target organelles of this toxin (Alonso-Garrido et al. 2018). A similar study design with BEA, showed that, besides oxidative phosphorylation and electron transport chain in mitochondria being the most significantly altered pathways, also apoptosis was affected (Escriva et al. 2018).

### Mixtures

*Fusarium* toxins are often found in combinations in grains and food which can alter their individual hazard and constitute a risk to consumers. However, only few in vitro studies addressed the effects of mixtures amongst ENNs (Table 2). Overall, these studies showed that combined effects of ENNs are dependent on the concentrations, but also on the type of combination and cell sensitivity. To the best of our knowledge, only one study is available focussing on BEA and ENN B in combination.

**Table 2:** Mixture studies with beauvericin and enniatins

Cell line	Toxicity endpoint; test concentration; exposure time	Mycotoxin combination	Mixture effect	References
Caco-2	Cytotoxicity (MTT): 0.625–5 µM (1:1) 1.25–2.5 µM (1:1:1) 1.25–2.5 µM (1:1:1:1) 24 h	ENN A + A1 ENN A + B ENN A + B1 ENN B + A1 ENN B1 + A1 ENN B + B1 ENN A + A1 + B ENN A + A1 + B1 ENN A + B + B1 ENN A1 + B + B1 ENN A + A1 + B + B1	Additive	Prosperini et al. (2014)
		ENN B + A1 ENN B1 + A1 ENN A + A1 + B	Synergistic	
		ENN B + B1	Antagonistic (at low conc.)	
CHO-K1	Cytotoxicity (MTT): 0.625–5 µM (1:1) 0.3125–2.5 µM (1:1:1) 24 h	ENN A + B1 ENN A1 + B ENN B + B1	Additive (all conc.)	Lu et al. (2013)
		ENN A + A1 ENN A + B ENN A1 + B1 ENN A + A1 + B ENN A + A1 + B1 ENN A + B + B1 ENN A1 + B + B1	Synergistic (at high conc.)	
		ENN A + A1 + B1 ENN A1 + B + B1	Antagonistic (at low conc.)	
HL-60	Cytotoxicity (flow cytometry with DAPI): 0.03–16 µM + 0.015–8 µM (2:1) 24h	BEA + ENN B	Synergistic	Juan-Garcia et al. (2024)
MDA-MB-231	Cytotoxicity (flow cytometry with DAPI): 0.03–16 µM + 0.015–8 µM (2:1) 24h	BEA + ENN B	Antagonistic	Juan-Garcia et al. (2024)
PBMC	Cytotoxicity (flow cytometry with DAPI): 0.03–16 µM + 0.015–8 µM (2:1) 24h	BEA + ENN B	Antagonistic	Juan-Garcia et al. (2024)
SH-SY5Y	Cytotoxicity (MTT): 0.1–10 µM (1:1) 24 h	ENN A + B1	Additive	Perez-Fuentes et al. (2022)
		ENN A + A1 ENN B + A1 ENN B + B1 ENN A1 + B1	Antagonistic	

*Caco-2* human colon adenocarcinoma, *CHO-K1* Chinese hamster ovary cells, *HL-60* human promyelocytic leukaemia, *MDA-MB-231* human breast cancer, *PBMC* human primary peripheral blood mononuclear cells, *SH-SY5Y* human neuroblastoma, *DAPI* 4′,6-diamidino-2-phenylindole, *MTT* 3-[4,5-dimethylthiazol-2-yl]-2,5 diphenyl tetrazolium bromide

Lu et al. (2013) investigated the cytotoxic interaction of binary and tertiary combinations of ENN A, A1, B and B1 in CHO-K1 cells. All combinations inhibited cell growth in a concentration-dependent manner. Binary combinations of ENN A or ENN A1 produced a marked cytotoxic effect as compared to the single exposures. While binary combinations such as ENN A + B1, A1 + B and B + B1 showed an additive effect within all concentrations tested, a synergistic interaction of combined ENN A + A1 + B, A1 + B1, A + A1 + B, A + A1 + B1, A + B + B1 and A1 + B + B1 was observed at higher concentrations. Thus, in binary and tertiary combinations of ENN A, synergistic effects occurred at high concentrations, while antagonistic interactions were detected at lower concentrations for ENN A + A1 + B1 and ENN A1 + B + B1. Interestingly, tertiary combinations did not induce stronger cytotoxicity in CHO-K1 cells compared to binary combinations (Lu et al. 2013). Prosperini et al. (2014) also evaluated the interaction effects of ENN A, A1, B and B1 combinations on the cytotoxicity of Caco-2 cells. They observed a synergistic effect on cell viability for the combinations ENN B + A1, B1 + A1 and A + A1 + B, while most other combinations showed additive effects at medium (IC_25_ and IC_50_) and high (IC_75_ and IC_90_) affected fractions, except for the lower (IC_5_) fraction and the ENN B + B1 mixture, which exhibited antagonistic effects. Overall, these interactions might occur, because at low concentrations, and considering their structural similarity, ENNs are competing for the same receptors, while at higher concentrations their effects are the sum of their individual effects (Prosperini et al. 2014). More recently, the combined effects of ENN A1, B1, A and B were also investigated on neuroblastoma SH-SY5Y cells. All mixtures resulted in an antagonistic effect, with exception of ENN A + B1 that produced an additive effect (Perez-Fuentes et al. 2022). Juan-Garcia et al. (2024) investigated the effects of a binary mixture of BEA and ENN B in HL-60, MDA-MB-231 and PBMC. While the combination in HL-60 cells led to a synergistic effect, an antagonistic effect was observed in MDA-MB-231 and PBMC. Regarding the transcriptional effects of combined exposure to BEA and ENN B (using qPCR to analyse 30 selected target genes), changes in the expression of genes codifying mitochondria-related proteins was also found. In addition, the combined effect of both toxins seemed to up-regulate the expression of genes related to the generation of reactive oxygen species (ROS) and down-regulate the expression of antioxidant-related genes, which might lead to production of oxidative stress in exposed cells (Escriva et al. 2019).

### In vivo studies

In rodents, feeding studies seem to indicate a high tolerance to the dietary intake of ENNs even at higher doses than the maximum levels found in feed. Similar findings have been also reviewed for BEA in mice (Gruber-Dorninger et al. 2017). Wistar rats fed for 28 days with a dose of 20.91 mg/kg b.w. ENN A per day revealed no histological or biochemical changes (Manyes et al. 2014), and in the follow-up study by Juan et al. (2014) the feeding of Wistar rats with 465 mg ENN A/kg feed revealed normal growth, no signs of illness and no significant changes in body weight and food intake over a period of 28 days. However, the authors noticed an exposure-related inhibition of the relative number of cytotoxic T lymphocytes (Juan et al. 2014). Another study on mice suggested sex-specific effects of ENN B and BEA, with males being more susceptible to oral exposure to BEA, while female mice seemed to be more susceptible to ENN B. In this study by Maranghi et al. (2018), mice were orally administered with ENN B (0.18, 1.8 or 18 mg/kg b.w. per day) and BEA (0.1, 1 and 10 mg/kg b.w. per day) over a period of 42 days. In males, the highest dose of ENN B led to an increased food consumption and a decreased body weight, and an increase in spleen weight and liver weight. In female mice, a decrease in body and liver weight at the lowest dose of 0.18 mg/kg b.w. per day was observed, as well as a decrease in kidney and thymus weight at doses of 0.18 and 18 mg/kg b.w. ENN B increased the brain weight in males of all treatment groups and in females treated with the highest dose. In contrast, BEA increased body weight of both sexes during the treatment period at the highest dosage. BEA also caused a decrease in liver weight (at 10 mg/kg b.w. per day), in mesenteric lymph nodes (at 1 mg/kg b.w. per day), in kidney (at 1 and 10 mg/kg b.w. per day), in thymus (at 1 mg/kg b.w. per day), and in heart (at 10 mg/kg b.w. per day) of male mice, while thyroid weight was decreased (at 1 mg/kg b.w. per day) in female mice (Maranghi et al. 2018). Different outcomes were obtained in a study by Ojiro et al. (2023) on ENN B, in which no clinical signs or alterations in body weight in any treatment group were observed, and the induced changes in food consumption were not dose related. These findings suggested a very low toxic potential of ENN B for mice at dose levels up to 30 mg/kg b.w. per day over a period of 28 days (Ojiro et al. 2023). Moreover, Rodriguez-Carrasco et al. (2016) also observed that a 5 mg/kg injection of ENN B or BEA in mice for 2 or 3 consecutive days did not cause reduced food and fluid consumption, fatigue or body weight alterations, and no signs of macroscopical or histopathological changes in the organs’ architecture (Rodriguez-Carrasco et al. 2016).

In vivo exposure to mixtures of ENNs have been approached in previous studies. A study conducted for 10 days in mice exposed twice a day to an intraoral administration of a 1% topical spray of fusafungine, showed low-grade dysplasia, fibrosis, hyperplasia, congestion, and oedema in the oropharyngeal mucosa, but without statistical significance (Yuca et al. 2006). Conversely, a repeated-dose 28-day oral toxicity study of an ENN complex (ENN B, ENN B1 and ENN A1) in mice reported a decreased food consumption in male (administered with 4 and 20 mg/kg b.w. per day) and in female mice (administered with 20 mg/kg b.w. per day), but no clinical changes or alterations in body weight were observed during the experiment, and no alterations in haematology, blood chemistry, or histopathology parameters were observed at the end of administration period (Okano et al. 2021). In Wistar rats, a single orally administrated dose of an ENN mixture (1.19, 2.16, 1.03 and 1.41 mg/kg b.w. for ENN A, A1, B and B1, respectively) also showed no adverse effects during 8h of the experiment (Escriva et al. 2015).

Concerning the effects of these mycotoxins on birds, most of the studies have been conducted on poultry. The EFSA CONTAM Panel identified, for broilers and laying hens, no-observed-adverse-effect levels (NOAELs) of 244 and 763 µg/kg b.w. per day for ENN B1 and NOAELs of 216 and 674 µg/kg b.w. per day for ENN B, respectively (EFSA 2014). Fraeyman et al. (2018a) investigated the impact of subchronic ENN B exposure (2352 µg/kg feed) on broiler chickens for up to 21 days. Histopathological analyses revealed that while no major abnormalities were found in the liver, proliferation of enterocytes in the duodenal crypts was inhibited, but the villus length, crypt depth, or villus length-crypt depth ratio of the jejunum and ileum were not affected (Fraeyman et al. 2018a). Furthermore, a study on broiler chickens exposed to contaminated feed has shown a strong positive correlation between the feed conversion ratio (FCR—ratio of total feed consumed to live body weight of chicken at the end of the trial) and the exposure to mixtures of ENNs (ENN A, A1, B and B1; coefficient of determination (*R*^2^) = 0.60) and BEA (*R*^2^ = 0.73), suggesting mycotoxins may negatively impact bird FCR, as increase in levels of toxin mixtures resulted in higher FCR (Kolawole et al. 2020).

In vivo effects on fish were approached by Berntssen et al. (2023) demonstrating that both ENN B (0.3, 5.2, 83 mg/kg feed) and BEA (0.3, 4.8, 46 mg/kg feed) were not able to affect bone formation as assessed from x-ray evaluation in farmed Atlantic salmon (*Salmo salar*), but caused a reduced specific growth rate, with a more pronounced effect in ENN B- (at medium and high exposure level) than in BEA-fed fish (only at the highest exposure level) over a period of 69 days and 76 days (Berntssen et al. 2023). In zebrafish (*Danio rerio*), exposure to ENN A, ENN B and BEA in egg water did not cause any anatomical phenotype, and the development and growth of zebrafish larvae were not affected. However, an increased percentage of dead larvae was observed with the increased concentrations of ENN A and ENN B dissolved in water, and a 100% death was reached with 25 µM of ENN A and ENN B at 24 h and 48 h exposure. BEA caused 100% dead larvae at the highest concentration (64 µM) at 24h, while at 8 μM and 72 h none of the larvae were dead. Concerning motility, all the three toxins impaired larvae motility, with ENN A showing the lowest values in motion followed by BEA and ENN B. When exposed to several combinations of these toxins (binary and tertiary), no alive larvae were recorded after 24 h and none evolved after 6 h of exposure (Juan-Garcia et al. 2021b). Other aquatic species were also assessed in recent studies. The IC_50_ values in *Daphnia magna* exposed to BEA and ENN B were determined by Juan-García et al. (2023). For BEA, the IC_50_ values ranged from 28 µM to 10.7 µM at 48 h and 168 h, respectively, and for ENN B from 12.5 µM to 4.5 µM at 48 h and 168 h, respectively. When in mixtures, the combination of low concentrations of BEA + ENN B (2 µM + 0.8 µM) reduced survival to 67%, while higher concentrations (8 µM + 1.6 µM) reduced to 52% after 96h of exposure. For both toxins individually, an increase in the offspring of *D. magna* was also noted; however, when in mixture, the combination of the highest concentrations caused a decrease in the offspring of 0.64-fold compared to the control. Moreover, BEA, ENN B and their mixtures also induced changes in the swimming patterns measured after 3h of exposure (Juan-García et al. 2023). BEA-induced toxic effects were also measured in *Caenorhabditis elegans*. In a study by Buchter et al. (2020), survival rates of 29.3, 33.2, 30.4 and 34% were obtained after a 48 h exposure to 100, 500, 750 and 1000 μM of BEA, respectively, while in controls a 96% survival was reached. The effects were also observed in the mean life span, with a reduction of 13% compared to the controls, and in animals, body size showing a dose-dependent reduction in the mean length and area compared to the controls. Progeny was also affected, showing a decline to 46.9, 47.9 and 37.1 larvae per nematode (vs. 59.3 larvae per nematode in controls) after exposure to 10, 50 and 100 μM, as well as animal locomotion, and their susceptibility to thermal stress. Median survival was reduced by 3.2, 10.8 and 14% with incubation with 10, 50 and 100 μM of BEA, respectively (Buchter et al. 2020).

### Summary

Evidence shows that ENNs and BEA can exert cytotoxic effects in multiple human in vitro models. The toxic concentrations vary between experiments, largely depending on experimental protocols including endpoints, cell types, exposure time, and the individual ENN analogue. In most studies, the cytotoxic effects of BEA and ENNs appear in the low µM range, with IC_50_ values in the range of 0.5 to 50 µM. The cytotoxicity increases with prolonged exposure time leading to lower IC_50_ values. Several comparative cytotoxicity studies have been conducted, yielding varied outcomes influenced by factors such as cell type, assay selection, and exposure duration (Ivanova et al. 2006; Meca et al. 2011a; Olleik et al. 2019; Prosperini et al. 2014). By comparing the cytotoxicity data amongst the most studied cell lines, it seems that lung cells a more susceptible compared to liver and intestinal cells. Among the ENN variants studied, ENN B emerges as the least toxic counterpart, while ENN A and ENN B1, followed by ENN A1 and BEA seem to exhibit higher cytotoxicity across multiple cell lines. However, more in vitro studies using metabolically active cell lines such as HepaRG cells or primary hepatocytes are needed. These cell types more closely mimic the livers in vivo conditions, especially in terms of detoxification and metabolisation capabilities. The combined effects of ENNs and BEA remain yet poorly addressed, but the available data suggest the occurrence of ENNs interactions that may enhance their effects in mixtures. The results from a limited number of mechanistic studies using transcriptomic approaches revealed that both ENN B and BEA negatively affect mitochondrial metabolism and cellular respiration and that their mixture has the additional potential of creating an imbalance of cell redox status. Furthermore, ENN B and ENN B1 were able to down-regulate genes related to several metabolic pathways of HepaRG spheroids, especially the steroid hormone biosynthesis pathway, that may impact the overall hormone production and that deserves further investigation in whole organisms. Although diverse toxic effects have been described in several different species, it is agreed that in vivo studies regarding toxic effects of ENNs and BEA are still scarce (Caloni et al. 2020; De Felice et al. 2023). The general toxic effects caused by these mycotoxins seem to be mild across different animal models, but higher susceptibility was observed in some species and sex-specific effects must also be taken into account.

## In vitro and in vivo genotoxicity

Genotoxicity may lead to adverse effects, including cancer, aging, and genetic diseases (if germ cell mutagenesis occurs), all of which are major concerns for human health. To ensure the safety of food-related compounds, the assessment of genotoxic effects is mandatory. Such assessments should evaluate the induction of gene mutations as well as chromosomal damage (i.e. structural and numerical chromosomal aberrations) (EFSA 2011, 2017). The available in vitro and in vivo studies investigating the genotoxicity and mutagenicity of ENNs and BEA are summarised in the next sections.

### In vitro studies

#### Bacterial cells

Fotso and Smith (2003) first investigated the mutagenicity of BEA in a bacterial reverse mutation assay using the *Salmonella typhimurium* strains TA97, TA98, TA100, TA102, and TA1535. BEA was found not to be mutagenic to these strains up to a concentration of 500 µg/plate. Moreover, the use of an external metabolising enzyme system (rat liver S9 fraction, up to 10%) had no impact on the number of revertant colonies induced by BEA. No toxicity data were provided for the tested *S. typhimurium* strains (Fotso and Smith 2003). The mutagenic potential of ENN B was assessed in TA98, TA100, TA102 and TA104 strains. ENN B and its metabolites (generated by rat liver S9 fraction) were not mutagenic up to the toxic concentration of 100 µM (Behm et al. 2009). Another study reported that ENN A1 and ENN B1 exert no mutagenic potential in the tested strains TA98 and TA100, both in the absence or presence of rat liver S9 fraction up to a concentration of 200 µM (Yilmaz 2014). No toxicity data were provided.

#### Mammalian cells

No mammalian gene mutation assays have so far been performed with BEA. In contrast, several studies have already demonstrated that BEA induces micronucleus (MN) formation in various cell lines (Table 3). Klarić et al. (2008) conducted a cytokinesis-block MN assay on PK15 cells, where they exposed the cells to different BEA concentrations (0.05, 0.5 and 5 µg/mL; corresponding to 0.064, 0.64, and 6.4 µM) for either 24 h or 48 h in the absence of S9 metabolic fraction. After 24 h, the MN frequency significantly increased at concentrations of 0.64 µM and 6.4 µM. Similar results were obtained in the 48 h exposure scenario, although the increase in MN frequency was only statistically significantly different from the negative control at the highest tested concentration (6.4 µM). It should be noted that cytotoxicity was not assessed in this study (Klarić et al. 2008). Celik et al. (2010) also reported an increase in MN formation in human lymphocytes after 48 h of exposure to 5 and 10 µM BEA in the absence of S9 metabolic activation. Within the same study, a chromosomal aberration test and a sister-chromatid exchange (SCE) assay were performed to examine the effect of BEA on human lymphocytes exposed to concentrations ranging from 1.25 to 10 µM for 48 h. Increases in the number of chromatid and chromosome breaks, fragments, numerical aberrations, and SCE were observed at all concentrations tested. It should be noted that a strong reduction of cell-proliferating ability (mitotic index) was observed at the two highest test concentrations of 5 and 10 µM (Celik et al. 2010). In another study by Juan-Garcia et al. (2019), HepG2 cells were exposed to different concentrations (0.3 to 2.5 µM) of BEA for 24 h in the absence of S9 metabolic fraction. A statistically significant increase in the MN frequency was present only at a concentration of 1.25 µM. Cytotoxicity was investigated by the authors in parallel assays. The IC_50_ value of BEA in HepG2 cells was estimated to be 7.01 µM after 48h of exposure, the time point at which MN formation was measured in this study (Juan-Garcia et al. 2019). Maranghi et al. (2018) also observed an increase in MN formation in undifferentiated HepaRG cells after 4h exposure to 6.3 µM BEA, while the next higher concentration of 12.5 µM was cytotoxic. In TK6 cells exposed to BEA for 3h (0.6–10 µM) or 24 h (0.3–5 µM), no effect on MN formation was observed either in absence or presence of rat S9 fraction. In the 3h condition, BEA was less toxic to the TK6 cells in the presence of S9 mix as reflected by the cytostasis values at 10 µM corresponding to 17% and 100% with and without S9 mix, respectively. When exposure was prolonged to 24 h, a cytostasis value of 79% was already observed at the 2.5 µM of BEA (Maranghi et al. 2018).

**Table 3:** In vitro genotoxicity and mutagenicity studies on beauvericin and enniatins

Assay	Cells, Test system	Exposure conditions (mycotoxin, concentration, duration)	Effects	References
		Beauvericin		
Alkaline Comet assay	Blood cells	0.1 and 0.5 µM 1 h, 24 h Standard alkaline comet assay	1 h: negative 24 h: positive at 0.5 µM (DNA damage) No toxicity data	Klaric et al. (2010)
	Caco-2	1.5—12 µM 24 h Standard alkaline comet assay	Positive at 12 µM IC_50_: 8.8 µM (NR), 20.6 µM (MTT)	Prosperini et al. (2013a)
	CHO-K1	0.1, 1 and 5 µM 24 h Standard alkaline comet assay	Positive at 1 µM Cell viability > 75%	Mallebrera et al. (2016)
	HEK293T	25 µM 24 h Standard alkaline comet assay	Negative No toxicity data	Tran et al. (2020)
	HL-60	20 µM 1 h Standard alkaline comet assay	Negative Cell viability > 90% (assay not specified)	Dornetshuber et al. (2009a)
	Jurkat-T	1.5, 3 and 5 µM 24 h Standard alkaline comet assay	Positive at 3 and 5 µM Cell viability > 60%	Manyes et al. (2018)
	KB-3–1	20 µM 1 h Standard alkaline comet assay	Negative Cell viability > 90% (assay not specified)	Dornetshuber et al. (2009a)
	PK15	0.1 and 0.5 µM 1 h, 24 h Standard alkaline comet assay	1 h: negative 24 h: positive at 0.5 µM (DNA damage) Cell viability > 82%	Klaric et al. (2010)
Bacterial reverse mutation assay	*Salmonella typhimurium* TA97, TA98, TA100, TA102 and TA1535	Spot test with single concentration of 2 µg/plate, ± S9	Negative	Fotso and Smith (2003)
		Plate incorporation test 0.2–500 µg/plate, ± S9	Negative	
		Preincubation test with single concentration of 20 µg/plate, + S9	Negative	
Chromosomal aberration test	Human lymphocytes	1.25–10 µM, – S9 48 h	Positive from 2.5 µM	Celik et al. (2010)
DNA intercalation assay	Salmon sperm DNA	50–150 µM 2 h	Weak DNA intercalation	Dornetshuber et al. (2009a)
Micronucleus assay	HepaRG (undifferentiated)	0.8–12.5 µM, – S9 4 h	Positive at 6.3 µM; cytotoxic at 12.5 µM	Maranghi et al. (2018)
	HepG2	0.312–2.5 µM 48 h	Positive at 1.25 μM	Juan-Garcia et al. (2019)
	Human lymphocytes	1.25–10 µM, – S9 48 h	Positive from 5 µM	Celik et al. (2010)
	PK15	0.05–5 µg/mL (6.4 nM to 6.4 µM) 24 h, 48 h	24 h: positive from 0.5 µg/mL 48 h: positive at 5 µg/mL	Klarić et al. (2008)
	TK6	0.625–10 µM, ± S9 3 h 0.312–5 µM, – S9 24 h	Negative Negative	Maranghi et al. (2018)
Sister chromatid exchange test	Human lymphocytes	1.25–10 µM, – S9 48 h	Positive at all doses	Celik et al. (2010)
γH2AX	HepaRG (undifferentiated)	0.01–25 µM 24 h	Positive ≥ 0.39 µM (cytotoxic)	Maranghi et al. (2018)
Topoisomerase I and II inhibition	supercoiled pGEM1 plasmid DNA	1 nM to 1 mM	Positive > 100 µM	Dornetshuber et al. (2009a)
	Enniatin A			
Alkaline Comet assay	Caco-2	1.5 and 3 µM 24 h Standard alkaline comet assay	Positive ≥ 1.5 μM (tail moment)	Prosperini et al. (2013b)
	HEK293T	25 µM 24 h Standard alkaline comet assay	Positive (tail intensity %)	Tran et al. (2020)
	Human lymphocytes	0.07–1.15 µM 1h	Positive ≥ 0.14 µM (tail intensity %), no concentration dependency	Mamur et al. (2018)
Chromosomal aberration test	Human lymphocytes	0.07–9.17 µM 24 h, 48 h	Negative	Mamur et al. (2018)
Micronucleus assay	Human lymphocytes	0.07–9.17 µM, – S9 48 h	Negative	Mamur et al. (2018)
Sister chromatid exchange test	Human lymphocytes	0.07–9.17 µM 24 h, 48 h	Negative	Mamur et al. (2018)
	Enniatin A1			
Alkaline Comet assay	Caco-2	1.5 and 3 µM 24 h	Positive at 3 µM (tail moment)	Prosperini et al. (2013b)
	HEK293T	25 µM 24 h	Positive (tail intensity %)	Tran et al. (2020)
Bacterial reverse mutation assay	*Salmonella typhimurium* TA98, TA100	Plate incorporation test 12.5 µM to 200 µM, ± S9	Negative	Yilmaz (2014)
	Enniatin B			
Alkaline Comet assay	Caco-2	1.5–3 µM 24 h	Negative	Prosperini et al. (2013b)
	HEK293T	25 µM 24 h	Positive (tail intensity %)	Tran et al. (2020)
	Jurkat-T	1.5, 3 and 5 µM 24 h	Negative	Manyes et al. (2018)
	RAW264.7	2.5 and 5 µM, ± FPG 24 h	Negative	Gammelsrud et al. (2012)
	V79	0.1–100 μM, ± FGP enzyme 3 h, 18 h	Negative	Behm et al. (2009)
Bacterial reverse mutation assay	*Salmonella typhimurium* TA98, TA100, TA102 and TA104	Preincubation test 100 nM to 100 µM, ± S9	Negative; Cytotoxic at 100 µM	Behm et al. (2009)
HPRT	V79	0.1–20 μM, ± S9 4 h	Negative; Cytotoxic at 20 µM	Behm et al. (2009)
Micronucleus assay	HepaRG (undifferentiated)	0.8–25 µM, – S9 4 h	Negative	Maranghi et al. (2018)
	TK6	0.625–10, ± S9 3 h 0.625–10, – S9 24 h	Negative Negative	Maranghi et al. (2018)
	V79	0.03–10 μM 18 h	Negative	Behm et al. (2009)
γH2AX	HepaRG (undifferentiated)	0.01–25 µM 24 h	Positive ≥ 1.56 µM; Inhibitory effects on cell proliferation ≥ 0.01 µM	Maranghi et al. (2018)
	Enniatin B1			
Alkaline Comet assay	Caco-2	1.5 and 3 µM 24 h	Positive at 3 µM (tail moment)	Prosperini et al. (2013b)
	HEK293T	25 µM 24 h	Positive (tail intensity %)	Tran et al. (2020)
Bacterial reverse mutation assay	*Salmonella typhimurium* TA98, TA100	Plate incorporation test 12.5 µM to 200 µM, ± S9	Negative	Yilmaz (2014)
	ENN mixture (3% A, 20% A1, 19% B, 54% B1)			
Alkaline Comet Assay	HL-60	20 µM 1 h	Negative	Dornetshuber et al. (2009a)
	KB-3–1	20 µM 1 h	Negative	Dornetshuber et al. (2009a)
DNA intercalation assay	Salmon sperm DNA	50–150 µM 2 h	weak DNA intercalation	Dornetshuber et al. (2009a)
Topoisomerase I and II inhibition	supercoiled pGEM1 plasmid DNA	1 nM to 1 mM	Positive > 100 µM	Dornetshuber et al. (2009a)

*Caco-2* human colon adenocarcinoma, *CHO-K1* Chinese hamster ovary cells, *HEK293T* human embryonal kidney, *HepaRG* human hepatoma, *HepG2* human hepatocarcinoma, *HL-60* human acute myeloid leukaemia, *Jurkat-T* human leukemic lymphoblasts, *KB-3-1* human cervix carcinoma, *PK15* porcine kidney epithelial, *RAW264*.*7* murine macrophages, *TK6* human chronic myeloid leukaemia, *V79* Chinese hamster lung fibroblasts, *MTT* 3-(4,5-dimethylthiazol-2-yl)-2,5-diphenyltetrazolium bromide, *γH2AX*: H2A histone family member X (form γ), *HPRT*: Hypoxanthine–guanine phosphoribosyl transferase

BEA has been tested with the in vitro comet assay in six studies. All of them were performed with the standard alkaline comet assay (no enzymatic detection of specific DNA lesions). Thus, only the induction of DNA strand breaks and alkali-labile sites was studied. First, BEA was tested in the HL-60 and the KB-3–1 cell line at 20 µM for 1h with no detectable changes in the comet tail intensity. The authors indicated that under these conditions cell viability was higher than 90% (Dornetshuber et al. 2009a). Klaric et al. (2010) tested BEA at 0.1 µM and 0.5 µM for 1h and 24h in PK15 cells and in blood cells of one human volunteer. In PK15 cells, statistically significant increases compared to control in the tail length, tail intensity and tail moment were observed at 0.5 µM after 24 h of exposure. Cell viability, measured with the 3-[4,5-dimethylthiazol-2-yl]-2,5 diphenyl tetrazolium bromide (MTT) assay under these conditions, was reported to be higher than 82%. In human blood cells, a statistically significant increase was observed at 0.5 µM after 24 h of exposure but only for tail moment. In this case, toxicity data were not reported (Klaric et al. 2010). In Caco-2 cells treated with BEA (1.5, 3 and 12 µM) for 24 h, only the highest concentration showed a statistically significant increase of the tail moment. However, cell toxicity under these conditions could have been high as the authors reported IC_50_ values of 8.8–20.6 µM in the neutral red uptake (NRU) and MTT assays, respectively (Prosperini et al. 2013a). BEA showed no statistically significant DNA damage, measured as % DNA in tail (i.e. tail intensity), in HEK 293T cells treated with 25 µM BEA for 24 h. It should be noted that no toxicity data were provided under the conditions tested in the comet test (Tran et al. 2020). In a more recent study by Mallebrera et al. (2016), the alkaline comet assay was employed using CHO-K1 cells treated with 0.1, 1, and 5 µM of BEA for 24 h. Among these concentrations, only 1 µM resulted in a statistically significant increase compared to the control. Under these experimental conditions, the IC_50_ value determined via the MTT assay was calculated to be 10.7 µM, with cell survival exceeding 75% at the 1 µM concentration (Mallebrera et al. 2016). Finally, in Manyes et al. (2018), a dose-dependent increase in the percentage of DNA in the comet tail was observed in Jurkat T-cells exposed to BEA at concentrations of 1.5, 3, and 5 µM for 24h, with the two higher doses showing statistically significant differences compared to the control. Cell survival, assessed using the MTT assay, was greater than 60% at 5 µM, 75% at 3 µM, and 100% at 1.5 µM (Manyes et al. 2018).

Maranghi et al. (2018) measured the ability of BEA to induce DNA double-strand breaks in undifferentiated HepaRG cells using the γH2AX (H2A histone family member X; form γ) assay. The treatment of cells with BEA at concentrations ranging from 0.01 to 25 µM for 24 h showed that γH2AX levels increased, but only at cytotoxic concentrations higher than 0.39 µM (Maranghi et al. 2018).

Dornetshuber et al. (2009a) demonstrated through cell-free assays that high concentrations (> 100 µM) of BEA can significantly intercalate into double-stranded DNA (dsDNA) and inhibit the catalytic activity of topoisomerase I and II (Dornetshuber et al. 2009a).

To our knowledge, there is so far only one study that examined mutagenicity (base pair mutations, frameshift mutations and small deletions and insertions) by ENNs in mammalian cells. In the study performed by Behm et al. (2009), the hypoxanthine–guanine phosphoribosyl transferase (HPRT) gene mutation assay was performed with ENN B in V79 cells. No increase in the gene mutation frequency was observed after 4h treatment with ENN B in the range of 0.1 to 20 μM, neither in the presence or absence of S9 metabolic fraction (Behm et al. 2009).

Few studies are available assessing the effects of ENNs on MN formation. Behm et al. (2009) did not observe an impact on the MN frequency in V79 cells after 18h treatment with ENN B in concentrations up to 10 µM in the absence of S9 metabolic fraction. Testing at higher concentrations is not to be considered relevant as in the NRU assay that was performed in the same study, an IC_50_ value of 4 µM was reported for ENN B after 48h in V79 cells (Behm et al. 2009). Similarly, 4h treatment with ENN B (0.8–25 µM) did not increase the MN frequency in undifferentiated HepaRG cells in the absence of S9. Cytotoxicity was not observed in this study at the highest tested concentration (25 µM). In the same study, no increase in MN formation was found in TK6 cells treated with 0.6–10 µM ENN B for 3 h and 24 h in the absence or presence of S9. Pronounced cytotoxicity was found after 24h treatment starting from 2.5 µM (Maranghi et al. 2018). In addition, Mamur et al. (2018) reported no effect on MN formation in human lymphocytes treated with concentrations up to 9.17 µM ENN A for 48 h. Cytotoxicity was observed at concentrations starting from 2.29 μM (Mamur et al. 2018).

ENN A, A1, and B1 induced DNA strand breaks in HEK 239T at 25 µM (no data for toxicity were provided) (Tran et al. 2020) and in Caco-2 cells even at tenfold lower concentrations (1.5 and 3 µM, non-cytotoxic concentrations) (Prosperini et al. 2013b) of each toxin after 24 h of exposure, using the alkaline comet assay. Furthermore, ENN A induced DNA damage in the comet assay in isolated human lymphocytes at much lower concentrations (≥ 0.14 µM) after 1h of exposure (Mamur et al. 2018). The authors stated that the observed DNA damage after treatment with ENNs might be related to oxidative stress, as increased ROS generation and lipid peroxidation were observed in treated Caco-2 cells (Prosperini et al. 2013b). Moreover, silibinin, which is known to have potent antioxidant activity and protects various cell lines from ROS, reduced the levels of DNA damage induced by ENN A, A1, and B1 in HEK 239T cells, indicating that ENN-induced genotoxicity may be associated with oxidative stress (Tran et al. 2020). In contrast, ENN B did not increase DNA damage in human Caco-2 cells (up to 3 µM, non-cytotoxic concentrations) (Prosperini et al. 2013b), Jurkat-T cells (up to 5 µM, non-cytotoxic concentrations) (Manyes et al. 2018), and RAW264.7 cells (up to 5 µM, close to LC50 values) (Gammelsrud et al. 2012) after 24 h exposure assessed in the alkaline comet assay. In addition, ENN B even at much higher concentrations (up to 100 µM) did not induce DNA damage in V79 cells after a short exposure time of 3 h or a longer period of 18h (Behm et al. 2009). No additional DNA damage was observed for ENN B after the addition of formamidopyrimidine [fapy]-DNA glycosylase (Fpg), an enzyme that converts 8-oxo-7,8-dihydro-2'-deoxyguanosine to single-strand DNA breaks, in treated V79 and RAW264.7 cells at short (3h) and prolonged (18h and 24h) exposures and low to high concentrations (0.1–100 µM) (Behm et al. 2009; Gammelsrud et al. 2012). In contrast, Tran et al. (2020) showed that ENN B at a concentration of 25 µM induced DNA strand breaks to a similar extent as ENN B1 after 24h exposure in HEK 239T; however, the authors did not provide data on the potential cytotoxicity of the concentrations tested (Tran et al. 2020).

A complex mixture of ENNs (3% ENN A, 20% ENN A1, 19% ENN B, and 54% ENN B1 homologues) did not increase the DNA damage levels (mean comet tail intensities) after 1 h treatment of HL-60 and KB-3–1 cells. However, the ENN mixture significantly reduced the H_2_O_2_-induced comet tail intensity of HL-60 cells indicating potential antioxidant activity (Dornetshuber et al. 2009a).

Contrary, in undifferentiated HepaRG cells ENN B (0.01 to 25 µM) induced γH2AX formation concentration-dependently starting from 1.56 µM, with a concurrent reduction of cell viability. The authors declare that the observed reduced cell viability was due to an inhibiting effect on the cell proliferation already beginning at the lowest concentration of 0.01 µM (Maranghi et al. 2018). ENN B also did not increase levels of γH2AX, as well as of p53 and p21 in RAW264.7 cells evaluated by Western blotting and flow cytometry (Gammelsrud et al. 2012).

ENN A did not induce chromosomal aberration or SCE in human lymphocytes at concentrations from 1.25 to 10 µM for 48h of exposure (Mamur et al. 2018).

In a cell-free assay, Dornetshuber et al. (2009a) demonstrated that a mixture of ENNs (3% A, 20% A1, 19% B, and 54% B1 homologues) intercalated substantially into dsDNA and inhibit the catalytic activity of topoisomerase I and II but only at high concentrations above 100 µM (Dornetshuber et al. 2009a).

### In vivo studies

There is only one comprehensive in vivo study in CD-1 mice addressing the genotoxic potential of BEA and ENN B. The approach by Maranghi et al. (2018) consisted of an acute in vivo genotoxicity study over a maximum period of 3 days and a combined repeated oral dose toxicity study with reproductive/developmental toxicity screening over 42 days performed according to OECD TG 422 (see details in Table 4). The acute genotoxic potential of BEA and ENN B was assessed using the Alkaline Comet assay according to OECD TG 489 in seven organs including liver, blood, duodenum, kidney, colon, spleen, and bone marrow and MN assay in bone marrow and colon of male CD-1 mice. BEA showed no acute genotoxic potential up to a dosage of 200 mg/kg b.w. The exposure to ENN B yielded an increase in tail intensity at the lowest dose of 50 mg/kg b.w. in kidney sections although no histopathological findings different from the control sections were observed by γ2HAX staining. Liver and bone marrow of ENN B exposed mice showed genotoxic effects in form of significantly increased tail intensity in the intermediate and high dose of 100 and 200 mg/kg b.w. However, no increase in micronucleated, mitotic or apoptotic cells was shown in the MN assay by either BEA or ENN B. For the assessment of sub-chronic genotoxicity, male and female CD-1 mice were exposed to BEA and ENN B for up to 42 days and different genotoxic endpoints were investigated by Alkaline Comet assay, Neutral Comet assay, MN assay and Pig-a gene mutation assay. BEA showed no genotoxic potential, except for an increased tail intensity in kidney and duodenum of male mice at the intermediate dose of 1 mg/kg b.w. and an increased tail length in ovary at the highest dose of 10 mg/kg b.w. ENN B exerts no genotoxic effects in vivo after repeated oral administration. Although a decrease in tail length was observed in kidney cells of female mice at 1.8 mg/kg b.w., the authors declare that this specific effect by ENN B is of minor biological relevance due to a lack of a crosslinking effect of ENN B leading to a decrease of tail length and an inappropriate protocol for measuring such effect (Maranghi et al. 2018).

**Table 4:** In vivo genotoxicity and mutagenicity studies of beauvericin and enniatins

Assay	Animal, organ/tissue	Route, dosage, duration	Effects	References
	Beauvericin			
Alkaline Comet Assay	Male CD-1 mice (*n = *5/group); liver, blood, duodenum, kidney, colon, spleen, bone marrow	Oral administration 50, 100 and 200 mg/kg b.w. per day 3 days	Negative	Maranghi et al. (2018)
	Male CD-1 mice (*n = *5/group); liver, bone marrow	Oral administration 50, 100 and 200 mg/kg b.w. per day 48 h	Negative	
	Male and female CD-1 mice (*n = *10/group); liver, blood, duodenum, kidney, ovary, testis	Oral administration 0.1, 1 and 10 mg/kg b.w. per day 42 days	Male mice: in kidney and duodenum increase in %TI at 1 mg/kg b.w Female mice: in ovary increase in tail length at 10 mg/kg b.w	
Neutral Comet Assay	Male CD-1 mice (*n = *5/group); epididymal sperm	Oral administration 0.1, 1 and 10 mg/kg b.w. per day 42 days	Negative	
Micronucleus assay	Male CD-1 mice (*n = *5/group); colon	Oral administration 50, 100 and 200 mg/kg b.w. per day 3 days	Negative	
	Male CD-1 mice (*n = *5/group); bone marrow	Oral administration 50, 100 and 200 mg/kg b.w. per day 48 h	Negative	
	Male and female CD-1 mice (*n = *10/group); blood	Oral administration 0.1, 1 and 10 mg/kg b.w. per day 42 days	Negative	
Pig-a gene mutation assay	Male and female CD-1 mice (*n = *10/group); blood	Oral administration 0.1, 1 and 10 mg/kg b.w. per day 42 days	Negative	
	Enniatin B			
Alkaline Comet Assay	Male CD-1 mice (*n = *5/group); liver, blood, duodenum, kidney, colon, spleen, bone marrow	Oral administration 50, 100 and 200 mg/kg b.w. per day 2 days	Kidney: increase in %TI at 50 mg/kg b.w	Maranghi et al. (2018)
	Male CD-1 mice (*n = *5/group); liver, bone marrow	Oral administration 50, 100 and 200 mg/kg b.w. per day 48 h	Liver: increase in %TI from 100 mg/kg b.w Bone marrow: increase in %TI at 200 mg/kg b.w	
	Male and female CD-1 mice (*n = *10/group); liver, blood, duodenum, kidney, ovary, testis, epididymis	Oral administration 0.18, 1.8 and 18 mg/kg b.w. per day 42 days	Male mice: negative Female mice: in kidney decrease in tail length at 0.18 mg/kg b.w. and increase of % hedgehogs at 1.8 mg/kg b.w. (the authors declare that this effect is of minor biological relevance); no results for blood due to damaged control blood sample	
Neutral Comet Assay	Male CD-1 mice (*n = *5/group); epididymal sperm	Oral administration 0.18, 1.8 and 18 mg/kg b.w. per day 42 days	Negative	
Micronucleus assay	Male CD-1 mice (*n = *5/group); colon, bone marrow	Oral administration 50, 100 and 200 mg/kg b.w. per day 48 h	Negative	
	Male and female CD-1 mice (*n = *10/group); blood	Oral administration 0.18, 1.8 and 18 mg/kg b.w. per day 42 days	Negative	
γH2AX assay	Kidney section of male CD-1 mice (*n = *5/group); kidney	Oral administration 50, 100 and 200 mg/kg b.w. per day 2 days	Negative	
Pig-a gene mutation assay	Male and female CD-1 mice (*n = *10/group); blood	Oral administration 0.18, 1.8 and 18 mg/kg b.w. per day 42 days	Negative	

*γH2AX* H2A histone family member X (form γ), *TI* tail intensity

### Summary

The few studies available do not provide evidence for the induction of gene mutations by BEA, ENN A, A1, B or B1. However, most of the assays performed in bacterial cells were not entirely done according to the respective OECD guideline (i.e., not all required five strains were used). Moreover, for several bacterial reverse gene mutation studies, the highest concentration tested was lower than the maximum concentration recommended by the OECD and no information based on overt cytotoxicity and/or precipitation was provided to justify the lower maximum concentration. A mammalian gene mutation study (i.e. the HPRT assay) was only performed for ENN B. Testing was done up to concentrations inducing clear cytotoxicity and no evidence was found for the induction of gene mutations. The results on the induction of chromosomal damage in vitro are not clear, due to contradictory findings. For BEA, increased MN formation was observed in different cell systems in the absence of a metabolic activation system, but not in TK6 cells. Moreover, in some studies, the increase in chromosome damage was associated with high cytotoxicity, whereas in other studies, the cytotoxic effects were not assessed. Overall, BEA yielded negative results in the comet assay at short exposure, while some studies reported the induction of DNA damage after 24h of exposure. However, it should be noted that two out of the four in vitro studies performing the comet assay only used a single concentration of BEA. Moreover, it should be further confirmed if the positive DNA damage response occurred at concentrations close to the IC_50_ (as shown by (Prosperini et al. 2013a)) or not (at concentrations inducing less than 20% cytotoxicity as shown by (Klaric et al. 2010)). Relating comet assay results to cytotoxicity is crucial, as highly cytotoxic concentrations may give rise to false positive results (Azqueta et al. 2022). Increased formation of chromosomal aberrations and sister chromatid exchanges, inhibition of topoisomerase I and II, weak intercalation in salmon sperm DNA and increased levels of γH2AX have also been reported after in vitro exposure to BEA. However, given the equivocal findings and the limitations within most of the studies, it remains unclear if BEA is genotoxic in vitro. The same holds true for the ENNs, although in general, less positive results were reported for these mycotoxins. ENN B and ENN A did not induce MN formation under any of the conditions tested and ENN A was also reported to be negative in the in vitro chromosomal aberration assay. In contrast, both ENNs as well as ENN A1 and B1, showed a genotoxic effect in one of the in vitro comet assays, although other in vitro comet assays with ENN B were negative.

In the latest EFSA opinion on the genotoxicity of BEA, the most recent in vitro studies in mammalian cell lines were considered as providing no convincing evidence for induction of chromosomal damage in the micronucleus and chromosome aberration tests or an increase of DNA strand breaks in the Comet assay (EFSA 2024), since no concentration-dependent effects were observed. Conversely, in vivo studies using the Comet and the Pig-a assays and the micronucleus test with BEA were negative, while equivocal results were observed in DNA damage in the duodenum and kidney, only seen at one dose level in one sex, leading to the assessment that BEA is devoid of genotoxic potential (EFSA 2024). On the other hand, the in vivo study results using ENN B support a genotoxic effect in bone marrow and liver cells after acute treatment, but not after repeated exposure (Maranghi et al. 2018). Overall, there are many data gaps concerning several toxins, and further testing according to current test guidelines is needed to conclude on genotoxic properties of these compounds.

## Immunotoxicity in vitro and in vivo

In recent years, selected studies have explored immunotoxic effects of mycotoxins, including ENN B and BEA, on diverse immune cell types using in vitro and in vivo studies. An increased recognition of the importance of immunotoxicity as a toxicological endpoint will enhance our understanding of the likely concentration-dependent immunomodulation and subsequent health effects such as increased susceptibility to infection as a result of immunosuppression.

### In vitro studies

#### Macrophages

Non-cytotoxic concentrations (≤ 2.5 µM) of ENN B induced cell cycle arrest in the murine monocyte-macrophage RAW264.7 cell line and increased the secretion of the proinflammatory cytokine interleukin (IL)-1β in lipopolysaccharide (LPS)-primed RAW264.7 cells (Gammelsrud et al. 2012). These effects were reduced by inhibitors of caspase 1 or cathepsin B, indicating that caspase 1 and the lysosomes were involved in ENN B-induced cell death and IL-1β release. The authors hypothesised that ENN B activates the inflammasome and IL-1β secretion by damaging the lysosome, as transmission electron microscopy images of ENN B-exposed cells showed damaged lysosomes. ENN B has also been shown to affect lysosomal stability and the release of cathepsin B from lysosomes to the cytoplasm in Caco-2 cells (Ivanova et al. 2012). In support of this, in mouse embryo fibroblasts, ENN B1 was found to destabilise the lysosomal membrane, resulting in leakage of chaperone-mediated autophagy-associated components (hsc70, hsp90, LAMP-2) into the cytosol (Oliveira et al. 2020). Comparable to murine RAW264.7 cells, also in human monocytic THP-1 cells, IL-1β expression was increased after co-exposure to LPS and ENN B (Korkalainen et al. 2017). In contrast to these more pro-inflammatory signals in already stimulated cells, another study showed that endocytosis decreased in macrophages that were exposed to 0.32 or 0.64 µM BEA or 1 µM ENN B during their differentiation from human umbilical cord blood (Ficheux et al. 2013). In accordance, ENN B (10 µM, 24 h) was also found to inhibit membrane-raft dependent endocytosis in RAW264.7 macrophages (Gammelsrud et al. 2012). Furthermore, ENN B did not affect monocyte to macrophage differentiation of THP-1 cells at lower concentrations (≤ 0.5 µM) (Solhaug et al. 2016). In a Transwell co-culture model with bovine mammary epithelial cells (MAC-T) and macrophages (BoMAC), epithelial cells were treated for 48h with 20% cytotoxicity-inducing concentrations of ENN B (29.9 µM) and BEA (11.3 µM). Analysis of cytokine secretion in the macrophage supernatants showed only decreases for IFN-γ, IL-8, IL-10, MCP-1 and IL-36RA (only BEA) after further 24 h with or without LPS treatment. However, the used concentrations were comparatively high, and no data are given about toxicity of the concentration to the macrophages (Shandilya et al. 2023).

#### Dendritic cells

Ficheux et al. (2013) demonstrated with long-term treatment (once for 1–6 days) in human umbilical cord blood samples that BEA and ENN B (1–2.4 µM) increase the secretion of anti-inflammatory IL-10. While ENN B decreased the expression of maturation markers such as *cluster of differentiation 80* (*CD80*), *CD86* and *C–C chemokine receptor type 7* (*CCR7*), BEA only decreased *CCR7* in dendritic cells from human umbilical cord blood (Ficheux et al. 2013). Noteworthy, decreased *CCR7* might affect the initiation of the adaptive immune response by interfering with dendritic cell migration (Schaeuble et al. 2012). In contrast, Yang et al. (2022) could stimulate the production of cytokine IL-12 and the expression of *CD86* in bone marrow-derived dendritic cells with BEA, although higher levels of BEA (2.5–10 µM) were tested for a shorter period (24 h) compared to the previous study. It was shown that these effects were mediated through toll-like receptor 4 (TLR4) (Yang et al. 2022). The involvement of TLR4 is consistent with reported findings of synergistic effects of ENN B with the known TLR4 agonist LPS in human THP-1 cells (Korkalainen et al. 2017) and RAW264.7 cells (Gammelsrud et al. 2012), as mentioned above. BEA (1.5 μM) alone did not affect the production of the immune signalling molecule IL-8 in Caco-2 cells after 24 h, but the secretion was increased in combination with DON (Albonico et al. 2016).

#### T lymphocytes

Apart for the already reported effects on macrophages and dendritic cells, BEA and ENN B were also reported to elicit responses in Jurkat-T cells, a human immortalised T lymphocyte cell line. Both mycotoxins were found to induce cell death (with BEA being more potent; see Table 1) and cause cell cycle arrest in S phase after 24 h of treatment (at 5 µM with BEA and between 3 and 5 µM for ENN B) (Manyes et al. 2018). In addition, BEA (1.5 to 5 µM) as well as a mixture of BEA and ENN B (0.1 to 0.5 µM) were able to alter the expression of several genes, mainly involved in mitochondrial pathways (Escriva et al. 2019, 2018), but also in genes linked to differentiation and immune response, including *IL-32* and *ABCG1*. Noteworthy, co-exposure of the Jurkat-T cells to both mycotoxins resulted in lower expression changes of *IL-32* and *ABCG1* compared to treatments with single mycotoxins (Escriva et al. 2019).

### In vivo studies

The existing body of research relating to in vivo immunotoxic effects of both, ENNs and BEA, draws from a small pool of studies.

For ENNs, there are two publications by Huang et al*.* in 2019 and 2022 looking at the effects of ENN B1 and ENN B in pregnant mice. ICR mice (*n = *20) were exposed daily for 4 days to doses ranging from 0 to 7 mg/kg b.w. for both ENNs. ENN B injection of female mice with 5 and 7 mg/kg b.w. per day led to a significant downregulation of chemokine (C-X-C motif) ligand 1 (*CXCL1*), *IL-1β*, and *IL-8* mRNA in foetal liver extracts (at day one), whereas for ENN B1 this was only the case at 5 mg/kg b.w. per day. Together, the studies show that these mycotoxins have the potential to modulate or even suppress embryonic innate immune function, which warrants further investigations into long-term effects on offspring resilience to infectious disease (Huang et al. 2019, 2022). In a 28-day subchronic study in Wistar Rats dosed with 465 mg ENN A/kg food (ad libitum, 28-day exposure), CD3 + T lymphocytes remained unchanged while the CD4 + T helper cell proportion increased, with a relative decrease by CD8 + cells (Juan et al. 2014). However, the EFSA CONTAM panel stated that the implications of these findings remain unclear (EFSA 2014). Maranghi et al. (2018) evaluated the immunotoxicity of ENN B in CD-1 mice dosed with 0.18, 1.8 and 18 mg/kg b.w. in a repeated dose 28-day oral toxicity study according to OECD TG 407. ENN B reduced IL-10 secretion and the number of CD3/CD8 + T cells, an effect that was only noticeable in male mice at 1.8 mg/kg per day but led to increased serum antibody levels (immunoglobulin (Ig) A and IgG) in females at this dose. The study by Maranghi et al. (2018) also evaluated immunotoxicity of BEA by dosing CD-1 mice with 0.1, 1 and 10 mg/kg b.w. per day over a period of 28 days. BEA had a more pronounced effect inapparent in female mice; particularly the increase of spleen T cell frequencies including CD3 + and double positive CD3 + /CD4 + lymphocytes and changes in cytokine production. Treatment with BEA for 42 days resulted in increased interferon (IFN)-γ and IL-10 at 10 and 0.1 mg/kg b.w., respectively. Conversely, the innate immune function, as measured by in vitro nitric oxide production from isolated adherent splenocytes, in response to LPS stimulation, was enhanced in male but not female mice (Maranghi et al. 2018). Further evidence of the immunotoxicity of BEA (4 mg/kg b.w. per day for seven days) was demonstrated in an experimental mouse colitis model whereby BEA decreased serum levels of tumour necrosis factor (TNF)-α and IFN-γ and induced apoptosis of activated T-cells. This immunosuppression led to an overall attenuated severity of colitis and was linked to inhibition of IFN-γ/STAT1/T-bet and downregulation of PI3K/Akt signalling (Wu et al. 2013).

### Summary

With regards to in vitro investigations of immunotoxicity, several studies on ENN B and BEA were found. However, no data is available for the other ENNs discussed in the present review. In addition to the cytotoxic effects on immune cells (see Sect. 3.1), an influence on immune cells at sub-cytotoxic concentrations has been observed in some studies. Also in vivo, immunomodulatory effects were found with ENN A, B, B1 and BEA in mice and rats. In conclusion, both ENN B and BEA showed immunomodulatory effects. The stimulatory or suppressive direction of these effects appears to be concentration- and toxin-dependent as well as on the co-stimulatory signals in vitro and gender in vivo.

## Endocrine effects

The endocrine system (ES) is composed of a group of structures called endocrine glands, responsible for the secretion of hormones. These glands play a vital role in numerous critical bodily functions, including growth and development, reproduction, electrolyte balance, immune response, and more. Due to the complex nature of the ES and the necessity for precise communication between organs, any disruption can have widespread consequences throughout the entire organism. In this context, we will specifically examine in vitro studies exploring the impact of ENNs and BEA on sex hormone receptors and steroidogenesis. It is worth noting that sex hormones indeed regulate the expression of many genes involved in central nervous system development, reproductive function, foetal growth, cell proliferation, the immune system, metabolism, and other processes. The identification and characterisation of endocrine disrupting activity exerted by ENNs and BEA, as well as their relative potency, would be highly relevant for their risk assessment.

### Effects on hormone receptors

Park and Lee (2021) investigated the agonistic and antagonistic effect of ENN A1 and ENN B1 on the human oestrogen receptor (ER) α by using the VM7Luc ER according to OECD TG 455 and human androgen receptor (AR) by using the 22Rv1/MMTV_GR-KO AR transcriptional activation assay according to OECD TG 458. No AR- or ER-mediated transcriptional activation was induced upon any concentration used (up to 1 µM or 10 µM, respectively) after 24h. On the other hand, ENN A1 and ENN B1 reduced the signal caused by the respective positive controls suggesting an antagonistic effect on ERα and AR. ENN A1 and ENN B1 had IC_50_ values of 0.914 µM and 1.03 µM on ERα and 0.765 µM and 0.813 µM on AR, respectively. To confirm that both mycotoxins are true antagonists of ERα and AR, a competitive binding assay was performed followed by studies on the induction of dimerization and translocation of ERα and AR by ENN A1 and B1. Although both ENN A1 and B1 were found to bind to ERα and AR, no receptor dimerization or nuclear translocation was induced. The authors concluded that the antagonistic effect could be attributed to the competitive binding of both mycotoxins to the receptors and subsequent blocking of the receptor dimerization which is necessary for the translocation to the nucleus (Park and Lee 2021).

ENN B-induced transactivation of ERα, AR, progesterone and glucocorticoid receptor has been tested in another study by using the human reporter gene cell lines MMV-Luc, TARM-Luc, TM-Luc and TGRM-Luc. It was shown that ENN B has no agonistic or antagonistic effect on the tested hormone receptors up to a non-toxic concentration of 1.56 µM (Kalayou et al. 2015).

In the same test system, Fernandez-Blanco et al. (2016b) examined possible receptor transactivation caused by BEA. No AR agonistic activity was induced after 48h up to a non-cytotoxic concentration of 1 µM. However, 1 µM BEA led to a significant reduction of the luciferase signals mediated by the progesterone receptor and the glucocorticoid receptor. As the next higher concentration of 10 µM was cytotoxic, the authors used High Content Analysis (measuring cell number, nuclear area, plasma membrane permeability, mitochondrial membrane potential and mitochondrial mass) to check for pre-lethal toxicity in the TM-Luc cell line. The authors confirmed the validity of the progesterone receptor antagonist response by BEA due to the lack of pre-lethal toxicity. BEA reduced also androgenic and estrogenic signals, but only at cytotoxic concentrations (10 µM) (Fernandez-Blanco et al. 2016b). In a reporter gene assay using plasmids expressing the human androgen and the marine sea bass oestrogen and avian thyroid receptor, BEA did not activate those receptors but had antagonistic effects on the androgen receptor and the thyroid receptor at 3.125 µM and 0.78 µM, respectively. In the androgen receptor cell model, BEA, however, induced cytotoxicity at the (next) highest dose tested in the cell viability assay (25 µM) (Garcia-Herranz et al. 2019).

### Effects on steroidogenesis

Studies describing adverse effects on steroidogenesis are available for ENN A, B and BEA, mostly on animal cell lines.

ENN B (0.01–100 µM) was able to reduce the level of testosterone, cortisol and progesterone, but not estradiol in H295R cells at concentrations higher than 10 µM. Mechanistically, the effect was confirmed by qPCR in H295R cells, demonstrating the repression of genes encoding for enzymes up-stream of the cholesterol synthesis (*3-hydroxy-3-methylglutaryl-coenzyme A reductase* (*HMGCR*)), or involved in the transformation of cholesterol in progesterone and pregnenolone (*steroidogenic acute regulatory protein* (*STAR*), *CYP11A*, *CYP17A1*). On the contrary, ENN B increased the expression of genes encoding enzymes downstream of the progesterone synthesis and controlling aldosterone and cortisol synthesis (*CYP21A2*, *CYP11B1*, *CYP11B2*), as well as estradiol synthesis (*CYP19A1*). ENN B also reduced estradiol levels in unstimulated porcine Leydig cells at 10 µM and inhibited the production of estradiol and testosterone in LH-stimulated porcine Leydig cells, but only at cytotoxic concentrations > 10 µM (Kalayou et al. 2015). Recently, a study reported that exposure of MA-10 Leydig cells to ENN B1, in the range 5–20 µM, determined a concentration–response decrease in progesterone and testosterone levels. In addition, in TM3 Leydig cells, ENN B1 at same dose regimen repressed gene and protein expression of enzymes involved in steroidogenesis (*Insl3*, *Hsd3b1*, *Cyp11a1*, *Cyp17a1*, *Hsd17b11*, *Star*), especially at the two highest concentrations. Such results should be taken with caution as at 10 and 20 µM ENN B1 decreased cell viability by over 40% (Shen et al. 2024). In addition, ENN A demonstrated to decrease the estradiol and progesterone secretion in small and large follicles of bovine granulosa cells in the range of 1 up to 5 µM, with a more pronounced effect on the small follicles (Chiminelli et al. 2022b). BEA decreased the secretion of both progesterone and estradiol in bovine granulosa cells at 3 µM; however, at the cytotoxic concentration of 10 µM, only progesterone levels were inhibited. As a confirmation, the expression of the *CYP11A1* and *CYP19A1* genes, encoding for the enzyme upstream progesterone and estradiol synthesis, respectively, were decreased by BEA treatment at 30 µM (Albonico et al. 2017). Conversely, an increase of *CYP11A1* expression was noted in porcine cumulus cells treated with BEA at a concentration of 10 µM even if a decrease in progesterone secretion was evident at the same concentration (Schoevers et al. 2016). A recent report confirmed the repression of progesterone and estradiol production by BEA on bovine granulosa cells at 6 µM (Spicer and Schutz 2022).

### Summary

Overall, the results from reporter gene studies were consistent, showing no nuclear receptor-mediated agonistic properties induced by ENNs and BEA up to the highest concentrations tested. On the other hand, ENN A1 and B1 were found to induce ERα and AR antagonistic effects at non-cytotoxic concentrations. BEA was also found to exhibit progesterone receptor antagonistic properties. Interpretation of the results regarding AR, GR and thyroid receptor antagonistic effects induced by BEA and ENNs is challenging as the decreased luminescence production was observed at or close to cytotoxic doses.

Concerning steroidogenesis, there is strong evidence indicating that BEA is able to reduce progesterone and estradiol synthesis in bovine granulosa cell systems. Available evidence on BEA effects in other steroidogenesis cell systems is sparce. In addition, ENN B has been shown to decrease testosterone, cortisol, estradiol and progesterone in different cell systems when very high concentrations where tested. ENN A has been also reported to decrease estradiol and progesterone production while ENN B1 decreased progesterone and testosterone levels but at cytotoxic concentrations. Although there is some evidence for disturbance of steroidogenesis, drawing any conclusions based on these limited observations would be quite uncertain, highlighting the need for further testing.

## Reproductive and developmental toxicity in vitro and in vivo

ENNs and BEA have been demonstrated to cause reproductive and developmental effects in several animal species by impairing the development of oocytes, deregulating granulosa cell steroidogenesis, impairing sperm function, and affecting testicular hormone synthesis amongst other effects (Chiminelli et al. 2022a). The capacity of BEA and ENNs to disrupt reproductive and developmental processes is described in detail in this section.

### In vitro and ex vivo studies

The effects of ENNs or BEA on ovarian function and early embryo development have been studied in several in vitro experimental models. In particular, BEA has been tested in porcine, sheep, gilt and sow oocytes and embryos, bovine granulosa cells as well as bovine ovarian cells. Oocyte and embryo function and development were impaired by BEA at concentrations as low as 2.5 μΜ (Albonico et al. 2017; Caloni et al. 2018; Mastrorocco et al. 2021, 2019; Perego et al. 2020; Schoevers et al. 2016, 2021). ENN B has been studied in mouse blastocysts and porcine embryos and was shown to impair embryo development at concentrations above 5 and 10 μM, respectively (Huang et al. 2022; Wang et al. 2021). The effects of BEA and ENNs on testicular function have been also studied in a number of in vitro studies. In a study using boar spermatozoa mitochondrial impairment was observed after treatment with BEA and a mixture of ENNs (Tonshin et al. 2010). In a similar model, ENN A, A1, B, and B1 inhibited boar sperm motility at concentrations ranging from 0.73 to 0.78 μM (Hoornstra et al. 2003).

### In vivo studies

Maranghi et al. (2018) examined the impact of BEA and ENN B on the reproductive/developmental system of male and female CD-1 mice and their offspring in a combined repeated-dose oral toxicity study according to the OECD TG 422. CD-1 mice (male, female and dams) were administered daily 0.1, 1 and 10 mg BEA/kg b.w. in olive oil or 0.18, 1.8 and 18 mg ENN B/kg b.w. in olive oil with 6% DMSO at 5 days per week over a period of 42 days. BEA led to a decreased absolute weight of the thyroid upon administration of the intermediate dose of 1 mg/kg b.w. per day, but only in female mice. In male mice, histopathological analyses revealed a colloid reduction in thyroid at 1 and 10 mg/kg b.w. per day, whereas pyknotic nuclei were increased in both male and female mice at the highest dose of 10 mg/kg b.w. per day. ENN B increased the number of thyroid follicles in male mice at the highest tested dose of 18 mg/kg b.w. per day. In female mice, the administration of BEA with 1 mg/kg b.w. per day led to an increase of endometrial cysts in uteri, while BEA at concentrations of 10 mg/kg b.w. per day induced endometrial hyperplasia. However, the lowest dose of BEA (0.1 mg/kg b.w. per day) induced a decrease of the myometrial area. ENN B increased both, the endometrial and myometrial area, when administered daily with 1.8 and 18 mg/kg b.w., and the 1.8 mg/kg b.w. per day dosing also increased the uterus luminal area. The administration of 10 mg BEA/kg b.w. per day to male mice resulted in a significant decrease in the percentage of unaffected tubules in testes. In fact, there was a significant increase in the percentage of atrophic tubules or tubules with disorganised germ cells at the same dose. Regarding hormone serum levels, BEA decreased serum testosterone levels in female and male mice upon 1 and 10 mg/kg b.w. per day, respectively. Conversely, ENN B decreased estradiol serum levels in male mice at 18 mg/kg b.w. per day. Daily BEA administration of 1 and 10 mg/kg b.w. and ENN B at 18 mg/kg b.w. resulted in a significant increase in serum T4 levels only in male mice, whereas female mice administered with ENN B at 18 mg/kg b.w. per day showed significantly lower thyroxine (T4) levels. Thyroid-stimulating hormone (TSH) levels were unaffected by both mycotoxins. Dams treated with 0.1 mg BEA/kg b.w. per day during pregnancy, including the day four post-partum, showed a decrease in absolute and relative weight of the thyroid. The administration of BEA at 1 and 10 mg/kg b.w. per day increased the degeneration of thyroid follicles. In addition, the highest administered dose decreased the number of follicles and follicular density in thyroid of dams. TSH serum levels were found to be increased in dams administered with 0.1 mg BEA/kg b.w. per day. The absolute and relative weight of ovaries in dams were increased upon administration of 10 mg BEA/kg b.w. per day. The treatment with BEA and ENN B showed no effect on the offspring, except a decreased weight of pups at post-natal day four (PND4) after administration of 1 mg BEA /kg b.w. per day to dams. According to the authors, a correlation with BEA administration is unclear (Maranghi et al. 2018). A study on male mice orally treated with 5, 10 or 15 mg/kg b.w. ENN B1 for 4 weeks observed a decrease in serum and intratesticular testosterone. Moreover, serum luteinizing hormone and gonadotropin-releasing hormone levels, as well as the activity of the testicular enzyme biomarkers lactate dehydrogenase and succinate dehydrogenase were decreased in a dose-dependent manner. The same study evidenced severe testicular damage, with alterations in germ cell arrangement, decrease of cellular layers and severe dilation of the seminiferous tubules, presence of exfoliated germ cells in the tubular lumen, as well as reduced number of spermatogonia, spermatocytes, spermatids, mature spermatozoa and Leydig cells. In addition, a reduction of sperm counts per cauda epididymis and sperm motility were also observed, in absence of alteration of testis weight and growth index (Shen et al. 2024). Okano et al. (2021) conducted a 28-day repeated-dose oral study in Crl:CD1 (ICR) mice and found that exposing the mice to a mixture of ENN B, B1 and A1 (at a ratio of 4:4:1) at 0.8, 4 and 20 mg/kg b.w. per day had no impact on haematology, blood biochemistry, or absolute weight of uterus, ovaries, epididymides, or testes. No changes in histopathology parameters of ovaries were noticed (Okano et al. 2021). Huang et al. (2019) used a mouse model to investigate the impact of ENN B1 on embryo development. When administering 1, 3, or 5 mg/kg b.w. per day ENN B1 for five days intravenously to female mice, increased apoptosis and inhibition of blastocyte proliferation were observed at the intermediate and high dosage, as well as embryo degradation and damage. The highest dose group (5 mg/kg b.w. per day) exhibited a significant decrease in foetal weight. Additionally, uterine content of female mice was evaluated 13 days after transfer of embryos derived from ENN B1 pre-treated blastocysts (1 to 10 μM). Transfer of embryos pre-treated with 5 and 10 μM ENN B1 to paired uterine horns of day four pseudo-pregnant mice, resulted in a decreased number of implantations and increased resorptions. The foetuses derived from blastocytes treated with 10 μM ENN B1 showed decreased survival and weight. No changes were observed in placental weight in the above procedure (Huang et al. 2019). A separate study conducted from the same group examined the effects of ENN B on early embryonic development. Intravenous injection of 1, 3, 5 or 7 mg/kg b.w. per day of ENN B resulted in a decreased total cell number of blastocysts, as well as induced apoptosis and necrosis in the two highest dose groups (5 and 7 mg/kg b.w. per day). Uterine content of female mice was assessed 13 days after transfer of embryos treated with varying concentrations of ENN B (5, 10, 20 or 40 μM). Results showed an increased ratio of implanted embryos that failed to develop and a decreased number of implantations in embryos pre-treated with 20 and 40 μM of ENN B. Blastocytes treated with 20 and 40 μM also exhibited decreases in placental weight, while pre-treatment with 10–40 μM of ENN B led to decreased foetal weight (Huang et al. 2022).

### Summary

Although further research is needed, accumulating evidence suggests that ENNs and BEA may negatively affect foetal development and reproductive functions. More specifically, BEA has been shown to affect oocyte and embryo development in several in vitro/ex vivo test systems. BEA has been also shown to have negative effects on the endometrium, ovaries, testis and to decrease serum testosterone after in vivo administration in mice. ENN B and B1 have been shown to impair embryo development and sperm parameters in a number of in vitro and in vivo test systems. In addition, ENN B was found to negatively affect the endometrium, uterus and circulating levels of estradiol when administered in mice. Finally, ENN B1 has been shown to decrease the number of implantations and to increase resorptions in mice, as well as to severely affect testis morphology. Further work is needed to bridge and establish the above-mentioned observations as well as to gather information on the remaining ENNs, for which only scarce data are available.

## Conclusion

The current understanding of ENNs and BEA reveals complex toxicological and toxicokinetic profiles, yet significant data gaps remain, impairing a comprehensive risk assessment for human health.

One of the major data gaps lies in the toxicokinetics of ENNs and BEA, especially in humans. The wide range of values observed for the bioavailability in animal studies indicates difficulties in determining the correct parameter, most likely due to problems of finding appropriate formulations for the i.v. and p.o. application of the lipophilic compounds. Thus, systemic exposure in humans is difficult to predict, and especially the significance of these findings for long-term human health remains unclear. It seems that ENNs and BEA undergo rapid distribution and extensive metabolism. A better understanding of excretion kinetics is also required, as current data are sparse.

In vitro studies consistently show that ENNs and BEA are cytotoxic to various cell types in the low micromolar range. This potency does not fully align with the results from in vivo animal studies, which are sparse and often report milder toxic effects. The discrepancies might be due to the limited intestinal absorption of ENNs and BEA, as observed in toxicokinetic studies. Moreover, the cytotoxic potential could be overestimated since ENNs and BEA undergo extensive biotransformation to more hydrophilic, presumably less toxic metabolites and the reported in vitro toxicity studies mostly used metabolically incompetent cell lines, which might have neglected the potential detoxification of ENNs and BEA.

The genotoxicity of ENN A, A1, B and B1 represents a significant area of uncertainty. While some in vitro studies suggest that exposure might cause DNA damage and chromosomal aberrations, the findings are inconsistent and in vivo genotoxicity studies have not adequately clarified these results. The availability of well-designed in vivo genotoxicity studies that adhere to regulatory guidelines is limited and thus represents a major gap.

Some data suggest that ENNs and BEA could have immunomodulatory and endocrine effects and interfere with reproductive functions. In terms of endocrine effects, data are mainly from in vitro studies, some with equivocal effects, and partially determined at concentrations close to cytotoxicity. However, the data are limited and yield inconsistent results so that their biological relevance is questionable.

The potential combined effects of ENNs and BEA in mixtures remain poorly understood, which is critical given that they regularly occur together in food and feed. Furthermore, comprehensive toxicological data on chronic dietary exposure are lacking, challenging an adequate risk assessment.

Overall, several critical data gaps were identified, highlighting the need to perform further studies on both BEA and ENNs. Since no manufacturer or producer, as for example in the case of industrial chemicals, is obliged to produce the information required for risk assessment, partners in the PARC project have undertaken the task of performing high quality experimental studies on ENN A, A1, B and B1, and BEA, covering toxicokinetics, genotoxicity, endocrine effects, and immunotoxicity, as well as other endpoints. This array of studies is ongoing, and results will be reported in due course. It is believed that the expected outcome of the initiated studies will improve the human health hazard characterization from exposure to ENNs and/or BEA. The data generated in PARC will be especially valuable for estimating the importance of climate change-related variations in mycotoxin occurrence patterns with regard to human health risks.
